# Identification of Benzimidazole Diamides as Selective Inhibitors of the Nucleotide-Binding Oligomerization Domain 2 (NOD2) Signaling Pathway

**DOI:** 10.1371/journal.pone.0069619

**Published:** 2013-08-01

**Authors:** David J. Rickard, Clark A. Sehon, Viera Kasparcova, Lorena A. Kallal, Xin Zeng, Monica N. Montoute, Tushar Chordia, Derek D. Poore, Hu Li, Zining Wu, Patrick M. Eidam, Pamela A. Haile, Jong Yu, John G. Emery, Robert W. Marquis, Peter J. Gough, John Bertin

**Affiliations:** 1 Pattern Recognition Receptor Discovery Performance Unit, Immuno-Inflammation Therapeutic Area, GlaxoSmithKline, Collegeville, Pennsylvania, United States of America; 2 Biological Reagents and Assay Development, Platform Technology Sciences, GlaxoSmithKline, Collegeville, Pennsylvania, United States of America; 3 Screening and Compound Profiling, Platform Technology Sciences, GlaxoSmithKline, Collegeville, Pennsylvania, United States of America; McGill University, Canada

## Abstract

NOD2 is an intracellular pattern recognition receptor that assembles with receptor-interacting protein (RIP)-2 kinase in response to the presence of bacterial muramyl dipeptide (MDP) in the host cell cytoplasm, thereby inducing signals leading to the production of pro-inflammatory cytokines. The dysregulation of NOD2 signaling has been associated with various inflammatory disorders suggesting that small-molecule inhibitors of this signaling complex may have therapeutic utility. To identify inhibitors of the NOD2 signaling pathway, we utilized a cell-based screening approach and identified a benzimidazole diamide compound designated GSK669 that selectively inhibited an MDP-stimulated, NOD2-mediated IL-8 response without directly inhibiting RIP2 kinase activity. Moreover, GSK669 failed to inhibit cytokine production in response to the activation of Toll-like receptor (TLR)-2, tumor necrosis factor receptor (TNFR)-1 and closely related NOD1, all of which share common downstream components with the NOD2 signaling pathway. While the inhibitors blocked MDP-induced NOD2 responses, they failed to block signaling induced by NOD2 over-expression or single stranded RNA, suggesting specificity for the MDP-induced signaling complex and activator-dependent differences in NOD2 signaling. Investigation of structure-activity relationship allowed the identification of more potent analogs that maintained NOD2 selectivity. The largest boost in activity was achieved by N-methylation of the C2-ethyl amide group. These findings demonstrate that the NOD2 signaling pathway is amenable to modulation by small molecules that do not target RIP2 kinase activity. The compounds we identified should prove useful tools to investigate the importance of NOD2 in various inflammatory processes and may have potential clinical utility.

## Introduction

Effective host defense against pathogens relies upon multiple families of pattern recognition receptors (PRRs) expressed either on the cell surface or within the cytoplasm. Together the PRRs activate the innate immune system in response to conserved pathogen-derived molecules such as nucleic acids and cell wall components. NOD2 is one of the best characterized cytoplasmic PRRs and is expressed primarily in antigen-presenting cells and certain mucosal epithelia [1]. It is comprised of two N-terminal CARD homotypic interaction/effector domains, a central nucleotide binding and oligomerization (NACHT) domain, and a C-terminal leucine-rich repeat (LRR) domain believed to be important for ligand binding. NOD2 is thought to undergo a conformational change upon binding the bacterial cell wall component muramyl dipeptide (MDP) to enable ATP binding, oligomerization and recruitment of the serine/threonine kinase RIP2 [2], [3]. This in turn leads to recruitment of additional effector kinases including TAK1/TAB1 and the formation of a poly-ubiquitinated signaling complex that stimulates canonical NF-κB and MAPK (p38, JNK) pathways [4], [5] leading to increased synthesis of pro-inflammatory cytokines and chemokines [6]–[8]. NOD2 increases inflammatory cytokine production by macrophages and dendritic cells synergistically with several members of the toll-like receptor (TLR) family of membrane-associated PRRs [9], [10]. Such co-activation has been shown to be necessary for an optimal adaptive (antigen-induced) immune response in T and B lymphocytes [11].

Given the central role of NOD2 in regulating innate immune signaling it is perhaps not surprising that mutations in this PRR are associated with chronic inflammatory and autoimmune diseases [12]. For example, “gain-of-function” mutations within the NACHT domain of NOD2 cause Blau syndrome (also known as early-onset sarcoidosis), a rare granulomatous disease characterized by arthritis, skin lesions and uveitis [13]. In addition, mutations within the LRR domain of NOD2 are associated with increased susceptibility to Crohn's disease [14]. Whether the Crohn's disease-associated NOD2 variants confer a gain- or loss-of-function remains controversial. Although the associations are less robust, variants in NOD2 and the closely related NOD1 have also been linked to a variety of other inflammatory conditions including adult-onset sarcoidosis and atopic diseases [15], [16]. Whether abnormal NOD2 signaling contributes to the pathogenesis of inflammatory diseases in which there is no mutation in NOD2 remains to be established.

The role of NOD2 in initiating innate immune responses, and its genetic association with inflammatory diseases, identifies NOD2 as a potential target for therapeutic intervention. To our knowledge the only compounds demonstrated to inhibit NOD2 signaling are the plant-derived polyphenol curcumin [17], and arene-chromium diterpenes based on the anti-inflammatory pseudopterosins from a sea coral [18]. However, curcumin additionally inhibits NOD1 and TLR4 induced NF-κB, and the activity of the arene chromium complexes towards NOD1 was not reported. Moreover, although a cell-based high-throughput screen (HTS) for small molecule NOD2 inhibitors has been conducted no selective compounds were identified (Reed, J.C. PubChem BioAssay summary report URL: http://pubchem.ncbi.nlm.nih.gov/assay/assay.cgi?aid=1579&loc=ea_ras). Screening large compound collections for NOD2 inhibitors using a traditional biochemical/receptor binding approach has not been feasible due to the difficulty in expressing and purifying large quantities of functional NOD2 protein. Consequently, we employed a cell-based screening approach to search for inhibitors of MDP-stimulated cytokines coupled with extensive selectivity profiling in NOD2-independent assays. We describe here the cellular activity and structure-activity relationship for a benzimidazole diamide series exhibiting highly selective inhibition of NOD2 signaling pathways.

## Materials and Methods

### Ethics Statement

The human peripheral blood used in these studies was obtained from normal healthy volunteers with informed written consent and approved by the GSK institutional review board.

### Reagents

The HEK293-hNOD1 (293/hNOD1), -hNOD2 and -hTLR2 cell lines were purchased and used under license from Cayla-InvivoGen. HCT116 cells were obtained from American Type Culture Collection (ATCC). HEK293T cells were obtained from the GSK internal repository for biological reagents. The NOD activators iE-DAP, Tri-DAP, MDP, MDP-rhodamine, the TLR2/6 ligand Pam_2_CSK4, single stranded RNA (ssRNA40) and the antibiotic Blasticidin S used for maintenance culture of stable cell lines, were all obtained from InvivoGen. BacMam recombinant baculoviruses encoding full-length wild-type human NOD1 and NOD2 cDNAs driven by the CMV immediate early promoter/enhancer were generated at GSK [19]. The small molecule RIP2 and IKK inhibitors used as control compounds in these studies were generated at GSK. The potencies of these compounds for their respective target human kinase are: RIP2 IC_50_ = 4 nM, IKK2 IC_50_ = 1 nM.

### Compound synthesis

Complete procedures for the synthesis of all NOD2 pathway inhibitors shown in tables are provided in Methods S1. Compounds were generated at GVK Biosciences.

### Screening and profiling assays

Inhibitors of MDP-stimulated IL-8 release were screened in a 1536-well assay whereby 293/hNOD2 cells (5000 cells/well) in DMEM high glucose medium supplemented with 0.1% FBS and 10 μg/mL Blasticidin S were stimulated with 30 ng/mL MDP (EC_80_) in the presence of 10 μM compound. After 24 hours the amount of IL-8 secreted into the medium was determined by homogeneous time resolved fluorescence (HTRF) assay by the direct addition of a 1:1 mix of europium cryptate and XL665-conjugated anti-IL-8 antibodies according to the manufacturer's instructions (Cisbio). Fluorescence was measured after 2 hours in a ViewLux imager using UMB-AMC excitation and 618–671 nm emission filters (Perkin Elmer). Subsequent assays for IC_50_ determination were performed in 384-well plates with 293/hNOD2, hNOD1 or hTLR2 cells (5000 cells/well) in the above medium and stimulated with the EC_80_ concentration of the appropriate activator/ligand (30 ng/mL MDP, 60 ng/mL iE-DAP, 3–10 ng/mL Pam_2_CSK4). Inhibition of TNFR1-induced IL-8 release was performed with 293/hNOD2 cells stimulated with 20 ng/mL (∼EC_95_) rhTNFα.

Compound activity on endogenous NOD1 and NOD2 receptors was determined by inhibition of either Tri-DAP or MDP-stimulated IL-8 release, respectively, using HCT116 human colon carcinoma cells. These cells have been demonstrated to express native functional NOD1 and NOD2 and respond to NOD activation with increased NF-κB activity and secretion of IL-8 [20]. The cells were maintained in DMEM high glucose medium supplemented with 25 mM HEPES and 10% FBS. For assay, cells were seeded into 96 well plates (90,000 cells/well) in DMEM containing 1% FBS and pre-incubated with compounds for 1 hour before addition of either 1 μg/mL MDP or 25 μg/mL Tri-DAP. The level of IL-8 released into medium after 24-hour incubation was determined by HTRF assay as described above and fluorescence measured on an Envision model 2102 multilabel plate reader (Perkin Elmer). Although the majority of compounds exhibiting any cytotoxic activity would be expected to be inhibitory in all assays and therefore eliminated during selectivity profiling, any remaining cytotoxic compounds were identified in the HCT116 assay by visual inspection of cultures at the end of the 24 hour treatment period.

Direct effects of compounds on RIP2 kinase activity was determined by autophosphorylation of full length human FLAG-6His tagged RIP2 expressed and purified from baculovirus. Enzyme was pre-incubated with compounds in assay buffer (50 mM HEPES pH 7.5, 25 mM MgCl_2_, 0.05%[w/v] CHAPS, 1 mM DTT and 0.05 mg/mL BSA) in 384-well plates, and then incubated with 10μM ATP for 2 hours at room temperature. The amount of ADP generated was measured using the ADP-Glo universal ADP detection assay for kinases (Promega) and luminescence quantified on a ViewLux imager.

The original hit compound identified as a selective inhibitor of the NOD2 pathway was profiled for inhibitory activity against 300 human kinases (Reaction Biology Corp). The compound was tested in duplicate at a single concentration (1 μM) and was pre-incubated with enzyme for 20 minutes prior to addition of 10 μM ATP.

### Viral transduction of HEK293T cells

Over-expression of NOD1 and NOD2 was achieved by transduction of HEK293T cells with either NOD1 or NOD2 BacMam recombinant baculoviruses. HEK293T cells were chosen for these experiments because they express NOD proteins at negligible levels and thus do not exhibit increased NF-κB activity in response to NOD1 or NOD2 activators [2], [21]. The effect of compounds on activator-independent NOD1/2 signaling was determined by 30 minute pre-treatment of cells (in 96-well plates seeded the previous day in DMEM high glucose with 10% FBS at 60,000 cells/well) followed by infection with NOD1 or NOD2 BacMam virus (MOI = 50). IL-8 was measured in conditioned medium after 24 hour incubation at 37^o^C. To determine the effect of compounds on NOD1/2 activator-dependent IL-8 secretion in BacMam transduced HEK293T, the cells were infected with virus for 6 hours (MOI = 50), and following the removal of virus the cells were cultured over-night. The next day medium was again replaced and cells pre-treated with compound for 30 minutes followed by stimulation with either MDP (30 ng/mL) or iE-DAP (300 ng/mL). IL-8 was measured in conditioned medium by HTRF after 24-hour incubation at 37^o^C.

### ssRNA transfection of 293/hNOD cell lines and real-time RT-PCR

Cells seeded in 96-well plates at sub-confluent density (60,000/well) in DMEM + 1% FBS were transfected for 6 hours with ssRNA40 (1.25–6.67 μg/mL) using lipofectamine 2000 (Invitrogen) and RNA/liposome complexes prepared in Opti-MEM1 medium (Invitrogen). Induction of IFNβ gene expression was measured by real-time reverse transcription PCR (Taqman). RNA was isolated from cell extracts in Trizol (Invitrogen) using the RNeasy kit (Qiagen), and mRNA quantified following reverse transcription and PCR amplification using Taqman reverse transcription and Taqman PCR core reagent kits, pre-formulated primer/probe mixes and ABI 7900 sequence detection system (Applied Biosystems). Expression of IFNβ (ABI primer/probe mix Hs01077958_s1) was normalized to 18S ribosomal RNA.

### Uptake of fluorescent-labeled MDP

HCT116 cells seeded onto glass cover-slips at low density were pre-treated with GSK669 or the endocytosis inhibitor chlorpromazine for 30 minutes before addition of 2.5 μg/mL MDP-rhodamine. Live cells were viewed periodically over 6-hours incubation at 37^o^C using a Nikon eclipse TE2000-E fluorescent inverted microscope fitted with a CCTV camera and rhodamine filter.

### Human monocyte isolation and culture

Monocytes were purified from heparinized human whole blood by centrifugation on Ficoll (Histopaque 1077, GE Healthcare) followed by Percoll gradients. Monocyte purity was >95% by morphological assessment of Wright's stained cytospins. Cells were seeded at 100,000 per well in 96-well plates in RPMI containing 10%HI-FBS, pre-incubated for 1 hour with compounds, and then stimulated with either 1 μg/mL MDP or 25 μg/mL Tri-DAP. The concentration of IL-1β, IL-6 and TNFα in medium samples collected after 24-hour treatment was determined using the MS6000 human pro-inflammatory-4 II tissue culture kit (Meso Scale Discovery). The IL-8 concentration was determined by HTRF.

### Western blotting

Confluent cultures of 293/-hNOD1 and -hNOD2 cells in 24-well plates were serum-starved over-night. The next day, cells were pre-incubated for 1 hour with compound and then stimulated for 1 hour with either 50 μg/mL Tri-DAP or 25 μg/mL MDP, respectively. Whole cell lysates were prepared in 1X cell lysis buffer (Cell Signaling Technology) containing additional inhibitors of aspartic, cysteine and serine proteases and aminopeptidases (protease inhibitor cocktail set III, EMD Biosciences) and inhibitors of multiple phosphatases (phosphatase inhibitor cocktail sets II and III, Sigma-Aldrich). The levels of phosphorylated and total p38, JNK, ERK1/2 and total IκBα were determined by immunoblotting of ∼20 μg protein per sample using the following primary antibodies: phospho-p38 MAPK (12F8, #4631), p38 MAPK (#9212), phospho-JNK (#9251), JNK (#9252) and IκBα (#9242) all purchased from Cell Signaling Technology, and phospho-ERK1/2 (sc-7383) and ERK1/2 (sc-94) antibodies from Santa Cruz Biotechnology.

## Results

### Identification of a selective inhibitor of the NOD2 signaling pathway

**Figure 1:**
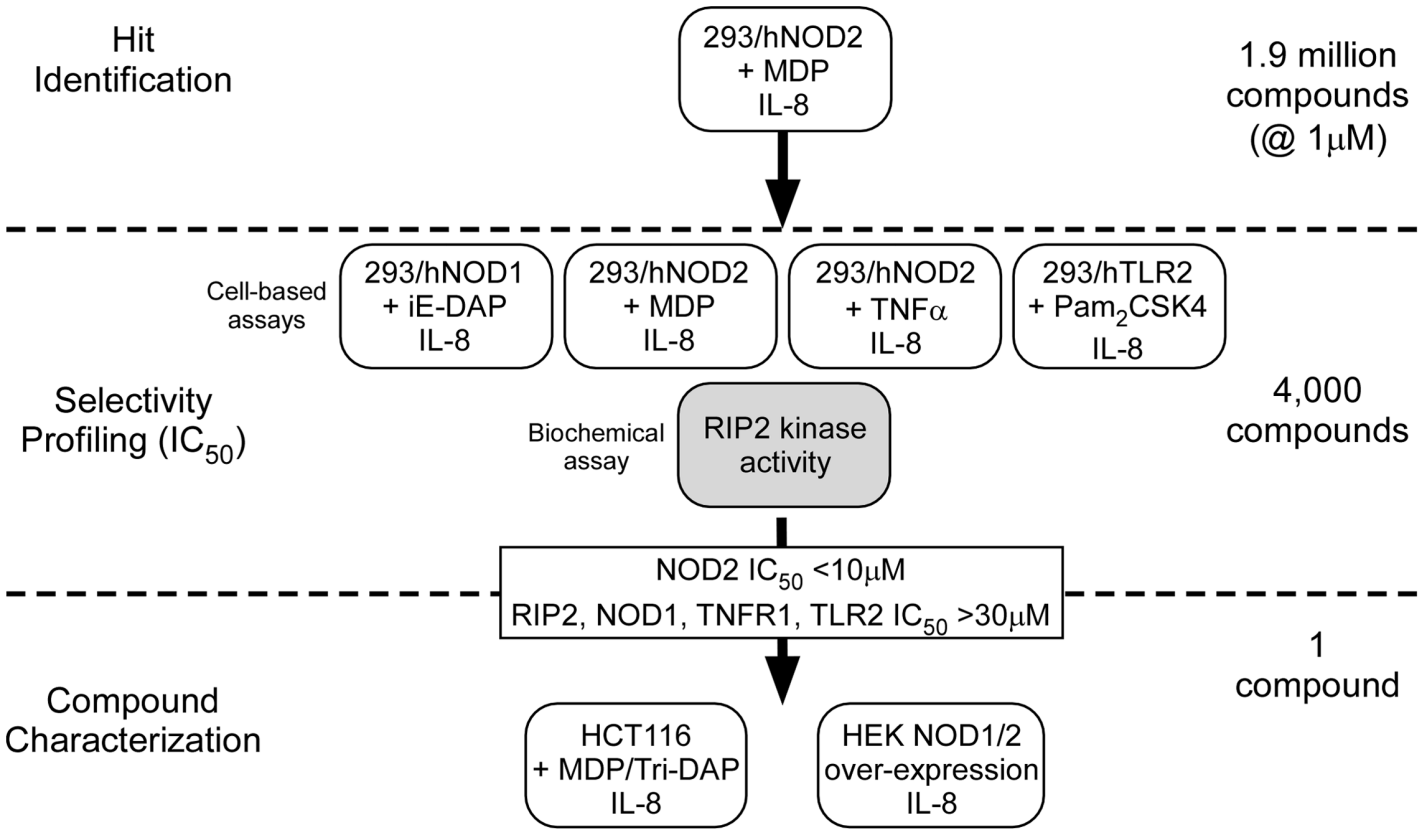
Cell-based screening scheme used to identify selective NOD2 inhibitors. The compound collection was screened for inhibitors of MDP-induced IL-8 secretion in 293/hNOD2 stable cells. Active inhibitors were then tested for selectivity against IL-8 induced by alternative pathways including NOD1, TNFR1 and TLR2 as well as for direct inhibitors of RIP2 kinase. The activity of selective NOD2 inhibitors was then confirmed in cells expressing NOD2 endogenously and against agonist-independent NOD2 signaling.

**Figure 2:**
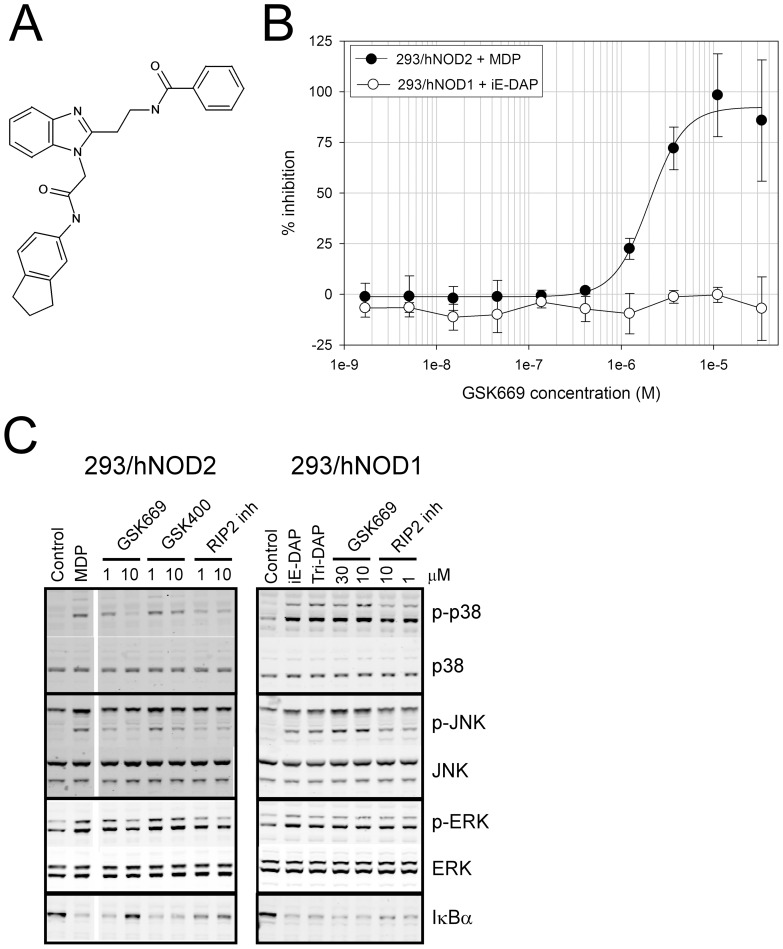
GSK669 inhibits NOD2 but not NOD1 mediated responses. (A) Structure of GSK669, the original hit identified from the NOD2 HTS screening cascade. (B) Concentration response curves showing GSK669 selectively inhibits MDP-stimulated IL-8. IL-8 secretion in either MDP treated 293/hNOD2 or iE-DAP treated 293/hNOD1 stable cell lines was measured in the presence of increasing concentrations of GSK669. Data are mean ± SD from 6 independent assays. (C) Selective inhibition of NOD2-mediated MAPK phosphorylation by GSK669. Serum-starved 293/hNOD1 or NOD2 stable cells were pre-incubated with compound at the concentrations indicated and then stimulated for 1 hour with either Tri-DAP or MDP, respectively. Phospho-p38, JNK and ERK1/2 were identified in cell lysates by western blotting. A RIP2 inhibitor compound was used as a positive control. Similar results were obtained in 5 (293/hNOD2) and 2 (293/hNOD1) separate experiments.

Selective inhibitors of NOD2 signaling were identified according to the strategy outlined in Figure 1. In brief, approximately 1.9 million compounds were screened for inhibition of MDP-stimulated IL-8 secretion in HEK293 cells stably expressing human NOD2 (293/hNOD2). Following confirmation of activity by repeat screening in duplicate, the active compounds were profiled in full dose-response for their ability to block MDP, iE-DAP, TNFα and Pam_2_CSK4-stimulated IL-8 release in the appropriate cell line to determine their inhibitory activity of the NOD2 mediated response relative to that mediated through NOD1, TNFR1 and TLR2, respectively. Our current understanding of the signaling pathways activated by these receptors suggests they converge at the level of the TAB/TAK1/IKK complex. Therefore, the selectivity assays served to identify compounds inhibiting shared components downstream of TAB/TAK1 as well as receptor-proximal upstream components unique to each pathway and not involved in NOD2 signaling. In addition, direct inhibitors of RIP2 kinase activity were eliminated by a biochemical assay measuring auto-phosphorylation of purified full-length human RIP2. This process identified a benzimidazole diamide, designated GSK669 (Figure 2A), as a selective inhibitor of the NOD2 pathway (IC_50_ = 3.2 μM). GSK669 was at least 10-fold less active in all of the selectivity assays and exhibited IC_50_ values >30 μM against NOD1, TNFR1 or TLR2 mediated IL-8 responses (Figure 2B, Table 1). GSK669 (at 1 μM) was also profiled for inhibition of kinase activity with a panel of 300 different human kinases, and none were inhibited more than 50% by the compound (Figure S1). A concentration of 1 μM GSK669 was chosen for kinase profiling because the activity of small molecules in cell-based assays is typically lower than in biochemical assays using purified protein, due to multiple factors including cell permeability, protein binding, metabolism and stability. Collection mining for similar structures to GSK669 revealed a second compound from this class, designated GSK400, that exhibited a similar activity and selectivity for inhibition of the NOD2-mediated IL-8 response (IC_50_ = 5 μM) showing that this activity was not unique to GSK669 (Table 1).

**Table 1:** Activity of NOD2 selective compounds and inhibitors of IKK and RIP2 in cell-based assays used for hit identification and triage.

	IC50 (μM)						
	NOD1/2 selectivity assays				Specificity assays		
Compound	293/hNOD1 iE-DAP	293/hNOD2 MDP	HCT116 Tri-DAP	HCT116 MDP	293/hNOD2 TNFα	293/hTLR2 Pam2CSK4	RIPK2 activity
GSK669 (**1**)	>50	3.2	>50	0.5±0.2	>100	>80	>100
GSK400 (**2**)	>50	5.0	>50	1.1±0.7	>100	>100	>100
RIP2 inh	0.006±0.001	0.008	0.004±0	0.002	>50	>10	0.0016
IKK inh	0.25±0.06	0.40±0.15	0.2±0.1	0.07±0.02	0.69±0.31	13.6±4.7	3.2

IC_50_ values are given in micromolar, as the mean and standard deviation. In each of the cell-based assays the end-point measured was IL-8. In the RIPK2 biochemical assay autophosphorylation of RIPK2 was determined.

### Selective inhibition of NOD2 versus NOD1-mediated NF-κB and MAPK signaling pathways

Recruitment of RIP2 to activated NOD2 results in the formation of a poly-ubiquitinated signaling complex, or nodosome, required for downstream activation of NF-κB and MAPK pathways [4], [8]. To determine whether GSK669 inhibited one or both of these pathways in a NOD2-selective manner the effect on total IκBα and phosphorylated p38, p-JNK and p-ERK1/2 levels was assessed in 293/hNOD1 and 293/hNOD2 cells stimulated with Tri-DAP or MDP, respectively. Time course analyses indicated that the phosphorylated MAPKs were maximally induced 45–60 minutes after addition of each NOD activator, with the weakest effects on ERK phosphorylation (data not shown). Pre-treatment of cells with GSK669 at 1 and 10 μM prevented MDP-induced increases in p-p38 and p-JNK and MDP-induced reduction in total IκBα in 293/hNOD2 cells. A small molecule inhibitor of RIP2 showed similar activity in these cells whereas GSK400 had more modest inhibitory effects (Figure 2C). Since MDP gave only weak induction of p-ERK the reduction of these levels by the small molecule inhibitors was more difficult to discern. In 293/hNOD1 cells, in contrast, only the RIP2 inhibitor prevented similar effects induced by Tri-DAP, whereas GSK669 even at 30 μM was inactive (Figure 2C). These findings demonstrate that GSK669 selectively inhibits both NF-κB and MAPK signaling pathways following activation of NOD2 but not NOD1.

**Figure 3:**
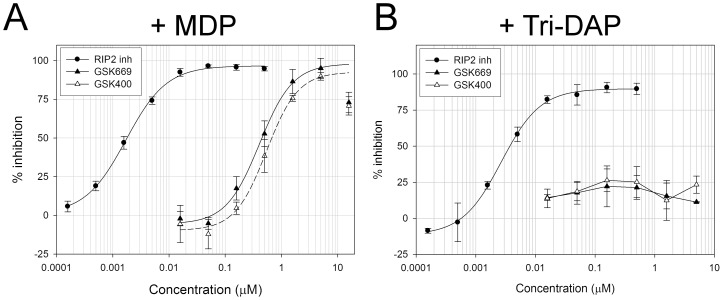
GSK669 exhibits NOD2 selectivity in cells that express endogenous NOD1 and NOD2. Concentration response curves of GSK669 and its close analogue GSK400 for the inhibition of MDP (A) but not Tri-DAP (B) stimulated IL-8 secretion in HCT116 cells which express functionally active NOD1 and NOD2. Cells were pre-incubated with compounds for 1 hour prior to addition of NOD agonists. IL-8 secreted into medium was assayed after 24 hours. The same RIP2 inhibitor used in Figure 2C was included as a positive control. Data are the average percent inhibition obtained from 1 (RIP2), 2 (GSK400) or 4 (GSK669) separate experiments.

The complete selectivity of GSK669 and GSK400 towards inhibition of NOD2 but not NOD1-mediated IL-8 secretion was also evident in HCT116 colon carcinoma cells which endogenously express both NOD1 and NOD2. Pre-treatment of these cells with compound for 1 hour dose-dependently inhibited IL-8 secretion induced by MDP (1 μg/mL) (Figure 3A) but had no effect on IL-8 secretion induced by Tri-DAP (25 μg/mL) (Figure 3B), whereas the RIP2 inhibitor antagonized the IL-8 response to both stimuli. The NOD2 compounds showed around a 5-fold higher activity in HCT116 cells compared to 293/hNOD2 stable cells, although the reasons for this are unclear. Table 1 summarizes the activity of the two NOD2 pathway inhibitors compared to RIP2 and IKK inhibitors in each of the assays described above.

### Optimization and SAR of GSK669 compound

**Figure 4:**
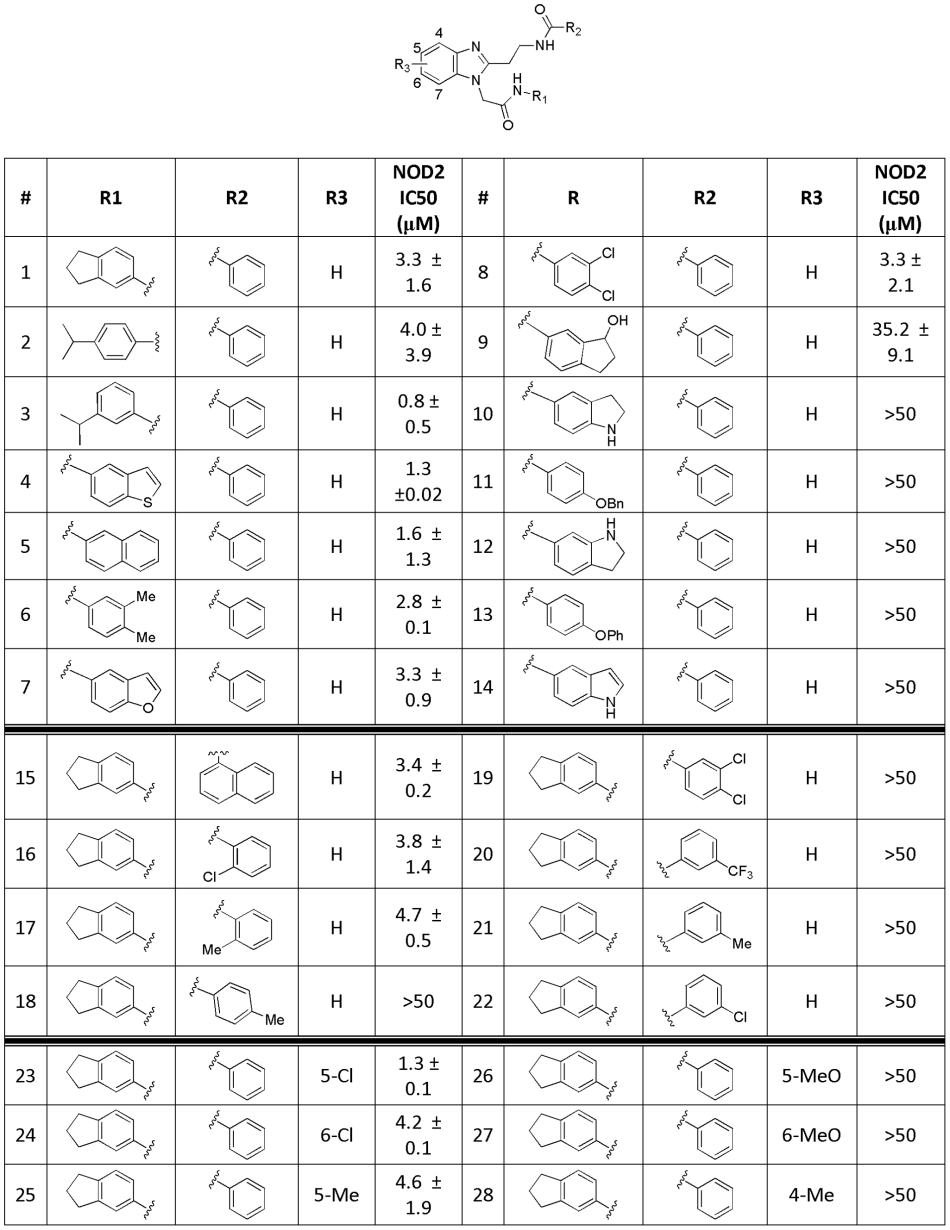
Structure-activity relationship for GSK669 analogs in the MDP-stimulated IL-8 production assay in 293/hNOD2 cells.

**Figure 5:**
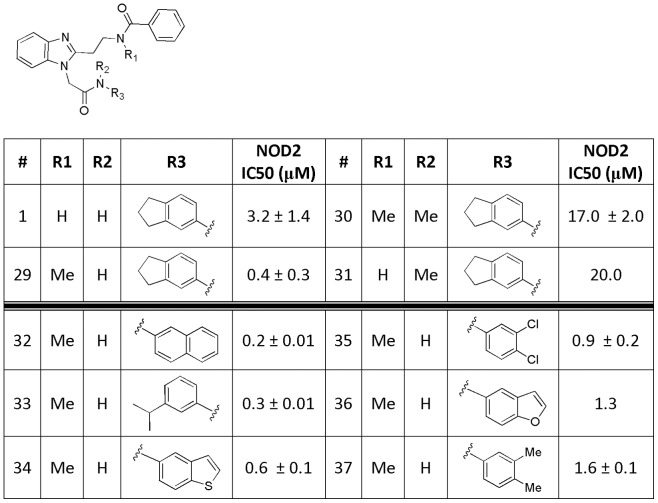
Structure-activity relationship for N-methyl analogs in the MDP-stimulated IL-8 production assay in 293/hNOD2 cells.

Upon confirmation of GSK669 and GSK400 (Figure 4, compounds **1** and **2)** as selective NOD2 inhibitors, additional analogs were designed, synthesized and tested for activity. The structure of the molecule allowed for rapid analog preparation. Both R1 and R2 groups were surveyed in parallel. Replacement of the dihydroindane (R1) proved to be the most tolerant of substitution. A wide array of groups are tolerated in this position with many of them having better activity than the original lead. However, R2 was less amenable to modifications allowing only substitution at the ortho position as demonstrated by compounds **16** and **17**. Additionally, a chloro substituent added to the benzimidazole core at both the 5- and 6- positions (Figure 4, compounds **23** and **24**) were allowed while all other changes were not tolerated. Conversion of the benzimidazole core to an unsubstituted imidazole resulted in a 5-fold loss of activity. The most significant improvement in activity came from methylation of the N-H amides. All possible combinations were prepared and tested including mono-methylation at both amides as well as di-methylation (Figure 5, compound **30**). Methylation of R1 (Figure 5, compound **29**) resulted in a 10-fold boost in activity resulting in the most active compound, GSK717. Other combinations caused a significant loss of activity. Finally, a small set of compounds were designed and prepared combining the most promising groups from Figure 4 with the N-methylation found in compound **29**. Compounds 32–37 (Figure 5) showed no additive effect and were essentially equipotent with compound **29**.

### GSK669 specifically inhibits MDP-induced NOD2 activation

**Figure 6:**
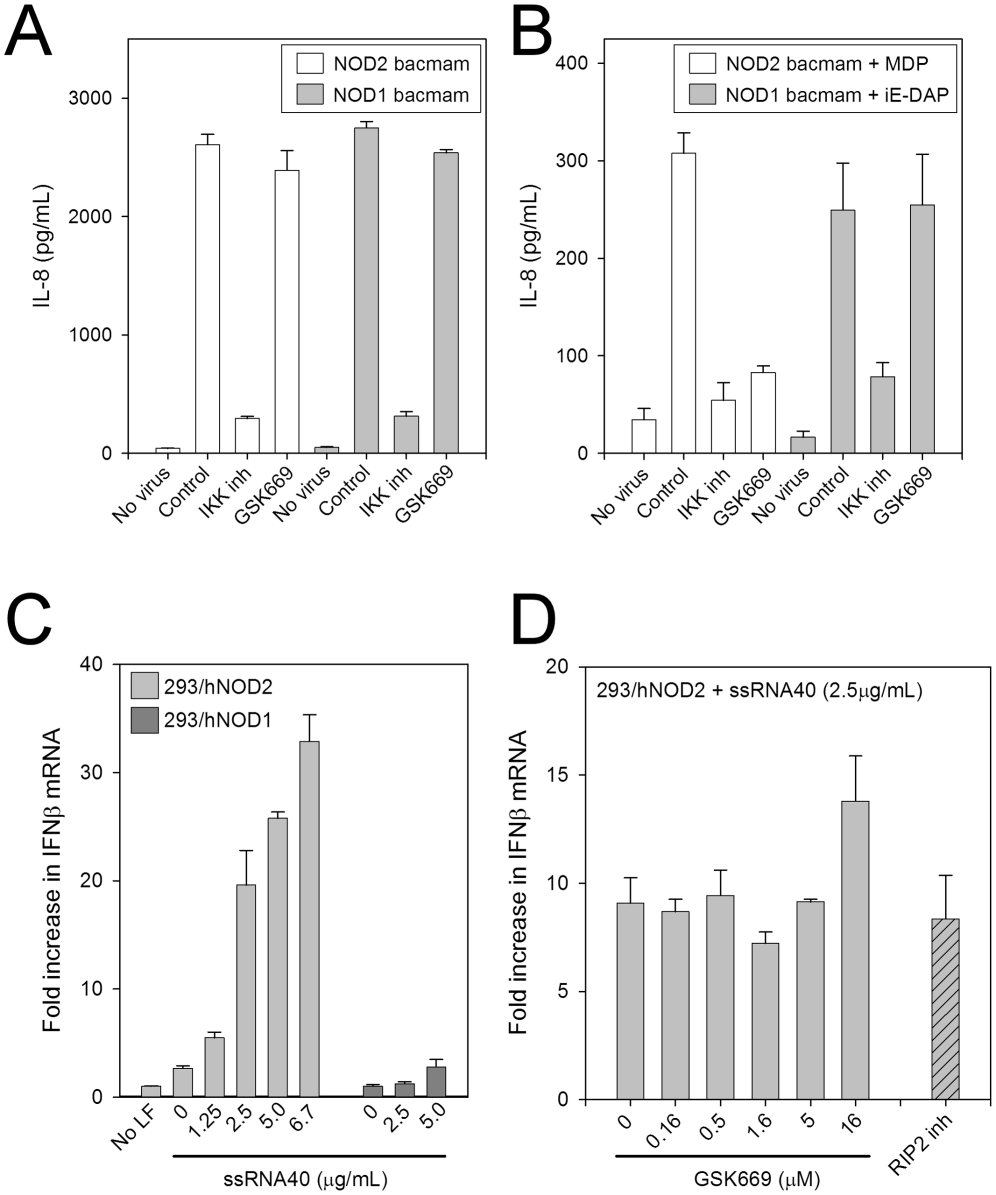
The activity of GSK669 is specific for inhibition of MDP-stimulated NOD2 responses. (A and B) Over-expression of NOD2 by viral transduction in HEK293T cells induces secretion of IL-8. Cells were infected (MOI = 50) with NOD1 or NOD2 bacmam virus alone for either 24 hours (A) or for 6 hours followed by removal of virus and stimulation with iE-DAP (300 ng/mL) or MDP (30 ng/mL) for an additional 24 hours (B). GSK669 (5 μM) failed to block IL-8 secretion induced by NOD1/2 virus alone but selectively blocked the MDP response in NOD2 transduced cells. The IKK inhibitor (0.5 μM) suppressed IL-8 release under all conditions. Data in (A and B) are the mean ± SD and are representative of two experiments. (C and D) Liposome-mediated transfection of ssRNA dose-dependently induces IFNβ gene expression in 293/hNOD2 but not 293/hNOD1 stable cells and the response was unaffected by GSK669 (0.16–16 μM) and a RIP2 inhibitor (0.1 μM). Cells were pre-incubated with compound for 30 minutes prior to transfection with ssRNA. IFNβ mRNA levels were measured after 6 hours by real-time RT-PCR. Data are presented as fold increase over mock transfected cells (mean ± SD) and are representative of two independent experiments.

In addition to activation by MDP, NOD2 has also been demonstrated to be activated by either over-expression induced self-oligomerization or by viral ssRNA [22], [23]. The ability of GSK669 to inhibit NOD2 signaling induced by MDP-independent mechanisms was therefore investigated. Although over-expression of NOD2 by viral transduction of HEK293T cells dramatically increased IL-8 secretion after 24 hours, treatment with GSK669 up to 50 μM failed to inhibit this response whereas a small molecule inhibitor of IKK blocked IL-8 secretion at the expected potency (Figure 6A). A RIP2 inhibitor also suppressed IL-8 release induced by NOD2 over-expression (data not shown). Transduction of HEK293T cells with NOD2 virus for a shorter time period (6 versus 24 hours) resulted in enhanced MDP-induced IL-8 secretion relative to the 24-hour transduction (Figure S2). This response to MDP was inhibited by GSK669 with a similar activity (IC_50_ = 0.5 μM) to that in the HCT116 colonic epithelial and 293/hNOD2 stable cell lines (Figure 6B, Table 1). In contrast, GSK669 had no effect on IL-8 secretion induced by over-expression of NOD1 either in the presence or absence of the NOD1 activator iE-DAP (Figure 6A and B).

Whereas MDP exposure and NOD2 over-expression primarily activate NF-κB and MAPK pathways, NOD2 activation by viral ssRNA stimulates a RIP2-independent, IRF3/type I interferon (IFNβ) pathway similar to that activated by TLR3 and RIG-I [23]. To mimic the NOD2 response to viral infection, 293/hNOD2 cells were transfected with ssRNA using liposomes with or without GSK669 pre-treatment. Transfection of ssRNA increased IFNβ mRNA expression in a concentration-dependent manner, although no increase in secreted IFNβ was detected (Figure 6C and data not shown). Consistent with the reported requirement for NOD2 but not NOD1 for the IFNβ response following ssRNA virus infection, only a minimal stimulation was observed in 293/hNOD1 cells. Pre-treatment of 293/hNOD2 cells with either GSK669 (0.16–16 μM) or RIP2 inhibitor (0.1 μM), however, did not block ssRNA-induced IFNβ expression (Figure 6D). Taken together, these findings suggest that GSK669 inhibits NOD2 signaling only when activated by MDP.

### NOD2 pathway inhibitors do not affect MDP uptake

Given the apparent selectively of GSK669 to inhibit MDP-induced NOD2 responses, we wanted to exclude the possibility that the compounds were blocking cellular uptake of MDP. The effect of GSK669 on uptake of Rhodamine-conjugated MDP by HCT116 cells was examined by fluorescence microscopy. Uptake of Rhodamine-MDP into cells resulted in the appearance of punctate structures in the cytoplasm over a 1–4 hour period which were unaffected by the presence of GSK669 up to 20 μM (Figure S3). In contrast, intracellular punctate fluorescence did not develop in cells treated with chlorpromazine, an inhibitor of clathrin-dependent endocytosis, in agreement with previous reports that MDP is primarily internalized by this mechanism [24].

### Inhibition of NOD2 stimulated cytokines in primary human monocytes

**Figure 7:**
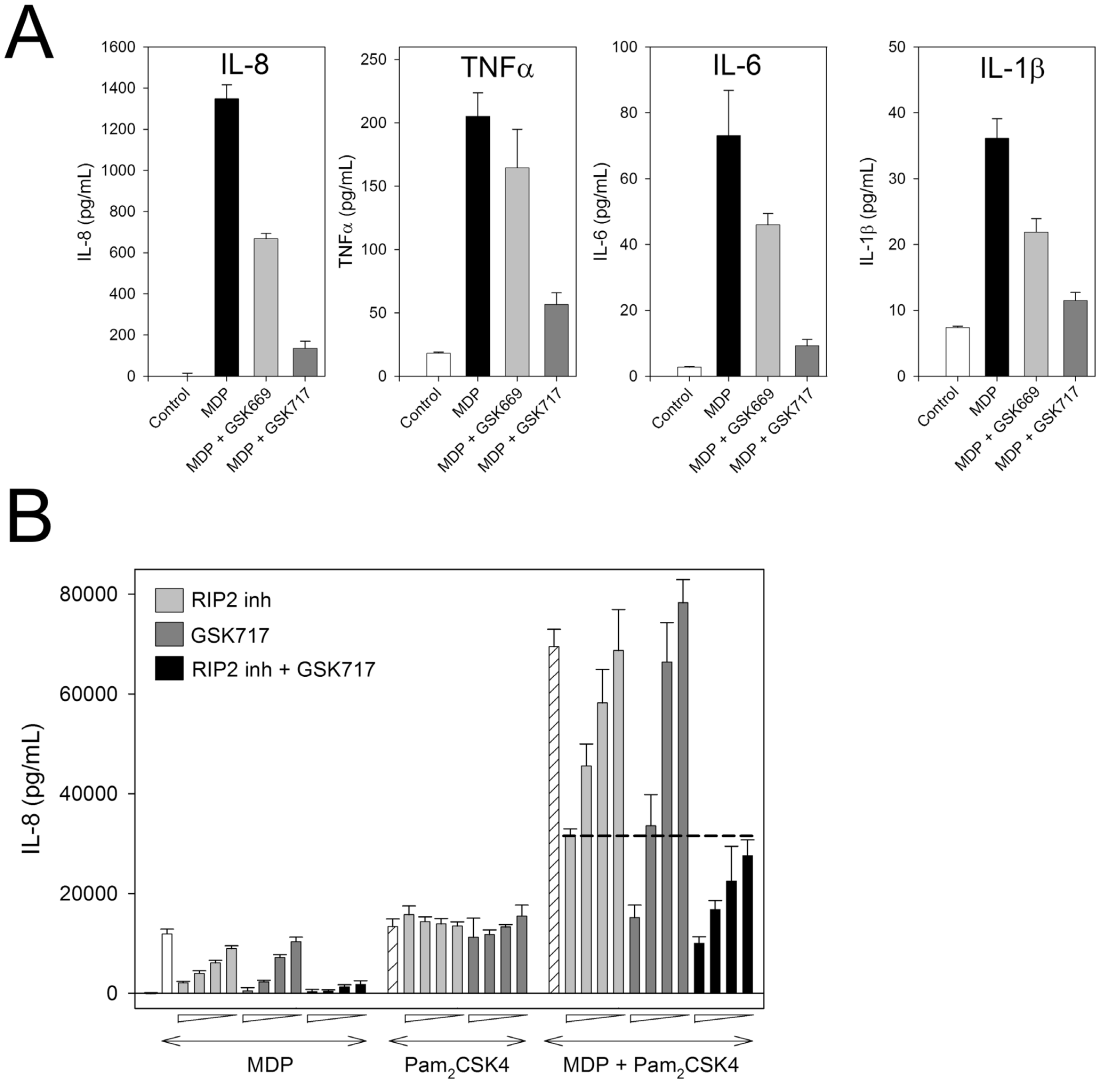
GSK669 and its analog GSK717 suppress cytokine secretion by primary human monocytes. NOD2 inhibitors suppress cytokine secretion in primary human monocytes activated via NOD2 alone as well as via synergistic NOD2-TLR2 co-activation. (A) Monocytes isolated from whole blood were immediately pre-incubated with NOD2 inhibitor compounds (5 μM) for 1 hour and then stimulated with MDP (0.1 μg/mL) for 24 hours. The concentration of IL-1β, IL-6, IL-8 and TNFα in the conditioned medium was determined. Data are mean ± SD of triplicate treatments from a representative experiment repeated once with similar results. (B) GSK717 blocks synergy between NOD2 and TLR2. Primary human monocytes were pre-incubated for 1 hour with either RIP2 inhibitor alone (0.05–1.6 μM), GSK717 alone (0.5–16 μM) or the combination of GSK717 (0.5–16 μM) and RIP2 inhibitor (at a fixed concentration of 1.6 μM) and then stimulated with MDP (0.1 μg/mL), Pam_2_CSK4 (100 pg/mL) or both agonists. IL-8 secreted into the medium was determined after 24 hours. The dotted line indicates the level of IL-8 released by co-stimulated cells treated with the highest concentration of RIP2 inhibitor. Results are representative of three independent experiments.

To determine the activity of the NOD2 pathway inhibitors in a primary human immune cell, we examined the effect on MDP-stimulated cytokine production by monocytes. GSK669 and active analogues dose-dependently inhibited the release of IL-8, IL-6, TNFα and IL-1β in primary human monocytes stimulated with MDP. At 0.1μg/ml MDP, GSK669 (5 μM) achieved partial inhibition while the more active analogue GSK717 (Figure 5, compound **29**) abrogated the stimulatory effect of MDP (Figure 7A). The rank order of compound activity matched that measured for MDP-induced IL-8 in HCT116 cells, irrespective of the cytokine, although the actual IC_50_ values indicated weaker activity compared to that measured in the cell line. Interestingly, inhibitor activity was inversely related to MDP concentration (0.1–10 μg/mL) suggesting a competitive interaction between MDP (or the direct NOD2 agonist) and GSK717 (antagonist) through binding to the same site on NOD2. To explore this possibility further, a Schild analysis was performed in which the MDP EC_50_ was determined in the presence of increasing concentrations of GSK717. Indeed, MDP dose curves were right shifted, and hence EC_50_ increased, with increasing GSK717 concentrations from 0.5 to 15 μM (Figure S4A). The Schild plot demonstrated a linear relationship between log(MDP dose ratio-1) and log(GSK717 concentration) with a gradient of 1.0, indicating that MDP and the inhibitor behave competitively (Figure S4B). While this finding is provocative, such relationships based on cell-based data need to be interpreted with caution when validated direct binding assays are unavailable.

### Inhibitors of NOD2 signaling pathway block NOD2-TLR2 synergy

Under most pathological conditions of infection and chronic inflammation it is likely that both NOD2 and multiple TLRs will be activated, a situation that often results in the synergistic stimulation of inflammatory cytokine release by macrophages and other responsive cells [9], [10]. We therefore determined to what extent the NOD2 pathway inhibitors could block a synergistic response in human monocytes co-stimulated with MDP and the TLR2/6 agonist Pam_2_CSK4. While both GSK717 (Figure 5, compound **29**) and the RIP2 inhibitor prevented MDP but not Pam_2_CSK4-stimulated IL-8 release, as expected, these compounds also abolished the clear synergistic IL-8 response from co-stimulated cells (Figure 7B). In co-stimulated cells, GSK717 (16 μM) exerted a greater maximal inhibition of IL-8 secretion than did the RIP2 inhibitor, reducing IL-8 release to the level induced by the TLR2 agonist alone. Moreover, treatment of co-stimulated cells with maximal inhibitory concentrations of both compounds had no additional inhibitory effect over GSK717 alone. These data suggest that the MDP/NOD2-mediated component of the synergistic response is partly RIP2 independent and that GSK717 is able to inhibit both the RIP2 dependent and independent components.

## Discussion

The association of NOD2 mutations with particular autoimmune and inflammatory diseases highlights its importance in innate immune regulation and infers that inhibition of NOD2 may represent a viable anti-inflammatory therapeutic strategy. However, few known components of the NOD2 signaling pathway represent attractive opportunities for pharmacological intervention. For instance, inhibition of RIP2 kinase–perhaps the most “druggable” component–blocks both NOD2 and NOD1 signaling, and identification of competitive inhibitors of ATP or agonist binding directly to NOD2 is hampered by difficulties in purification of NOD2 protein. Consequently, to our knowledge, selective NOD2 inhibitors have yet to be developed. The identification of the benzimidazole diamide series by a cell-based screening approach as described here, therefore demonstrates that the NOD2 pathway can indeed be selectively inhibited. The initial hit compound (GSK669) and its related analogues, GSK400 and GSK717, possess sub-micromolar in vitro activity and appear to exhibit significant selectivity for inhibition of NOD2-mediated responses since they do not block any of the other IL-8/NF-κB inducing pathways investigated including NOD1, TNFR1 and TLR2. Although the primary HTS utilized HEK293 cells stably expressing either exogenous NOD1 or NOD2, compound selectivity for inhibition of NOD2 signaling was maintained in immortalized and primary cells possessing endogenous functional NOD1 and NOD2 proteins.

Although the compounds are selective inhibitors of NOD2 signaling, the identity of their target protein and mechanism of action are unknown. These compounds did not inhibit the activity of RIP2 kinase or greater than 300 additional kinases. Moreover, the compounds lacked a typical activation loop/hinge-binding motif lending additional support to the conclusion that they are unlikely to be acting directly as kinase inhibitors. The data demonstrating lack of effect of GSK669 on cellular MDP uptake as well as on “ligand” independent activation in NOD2 over-expression systems suggest interference by the compound somewhere between intracellular processing of MDP and NOD2 oligomerization. Interestingly, these compounds blocked MDP-induced responses, but did not inhibit cytokine production induced by either NOD2 over-expression or single-stranded RNA. Given that NOD2 is the only known component shared between the MDP and ssRNA-induced NOD2 signaling pathways, the inability of GSK669 to block the response to ssRNA may imply that NOD2 is not its target. An alternative explanation of this finding, however, is that NOD2 activation by each process incurs subtle mechanistic differences. Thus, MDP, ssRNA, or expression-induced NOD2 oligomerization may each induce distinct NOD2 conformations, oligomeric structures, requirements for nucleotide binding, or complexes with other proteins, with the inhibitors possessing specificity for the MDP-induced mechanism since this was how they were originally selected in the primary screen. Our data suggesting a competitive interaction between MDP and the inhibitor GSK717 also implies engagement of NOD2. Lastly, other proteins identified as components of the NOD2 signaling complex, such as GRIM19 and TRIM27, may represent potential targets for GSK669 activity [25], [26]. Consequently, given that our knowledge of the exact composition of the NOD2 signaling complex or “nodosome” is incomplete, there are likely numerous other potential mechanisms for selective inhibition. Difficulties in expressing and purifying functional NOD proteins had made molecular approaches to examining inhibitor action largely unfeasible, but recent reports of advances in generating full-length NOD1 and NOD2 [3], [27] may enable assessment of direct interaction with NOD2 and effects on its biochemical activity. However, to date our own laboratories and that of an external collaborator have been unable to generate functional full-length NOD2 protein in addition to multiple other NLR proteins.

Other small molecule inhibitors of NOD1/2 signaling have been reported. Arene-chromium diterpene complexes were reported to be selective inhibitors of NOD2 based on their ability to inhibit NF-κB activity induced by MDP but not by agonists of TLR2, TLR4 or TNF receptor, or that resulting from RIP2 over-expression [18]. However, the selectivity profile of these complexes is incomplete since effects on NOD1-mediated responses and MAPK signaling were not reported. The benzimidazole diamide class of NOD2 inhibitors is both more potent and more chemically tractable compared to the arene-chromium diterpene. In addition, these inhibitors are not structurally related to either the arene chromium diterpene complexes or to the polyphenol curcumin, the latter exhibiting comparatively poor selectivity by inhibiting NOD1, NOD2 and TLR4 signaling [17], [18]. Two distinct classes of selective NOD1 inhibitors have been identified from a cell-based HTS for agonist stimulated NF-κB activity in cells possessing endogenous NOD1, namely 2-aminobenzimidazoles (PubChem ML-130) [28], [29] and purine-2,6-diones (PubChem ML-146, Magnuson et al, 2010, High throughput screening assays for NOD1 inhibitors–Probe 2. Probe Reports from the NIH Molecular Libraries Program [Internet]; National Center for Biotechnology Information (US): Bethesda, MD, 2010. PubMed PMID: 21433392, available from http://www.ncbi.nlm.nih.gov/books/NBK50701/). The 2-aminobenzimidazole NOD1 inhibitors were suggested to cause conformational changes in purified NOD1 protein (by compound ^1^H-NMR spectra) but not to affect ATP binding (by fluorescence polarization using FITC-ATP) nor interaction with RIP2 and certain other known associated proteins (by co-immunoprecipitation of exogenously expressed proteins) [28]. Again, an analysis of interference in these processes by the inhibitors we have identified may be possible due to the recent reports of the generation of functional NOD2 protein.

The ability of GSK669 and its more potent analogs to inhibit NOD2 signaling without affecting NOD1, TNFR1 or TLR2-mediated responses, renders these compounds suitable chemical tools with which to explore the contribution of NOD2 to various physiological and pathological inflammatory processes using in vitro and ex vivo cell and organ culture systems. For instance these compounds may prove useful for investigating the importance of NOD2 activation in the responses of intestinal, airway, reproductive and urinary tract epithelia and keratinocytes to pathogenic and nonpathogenic stimuli. The results of such studies should better define the clinical utility of these novel inhibitors in innate immune-driven human diseases.

## Supporting Information

Figure S1 Kinome plot showing the lack of kinase inhibitory activity for GSK669. GSK669 was screened at 1μM for inhibition of approximately 300 different kinases (Reaction Biology Corp.). The percent inhibition of kinase activity is color-coded for each kinase as indicated. (TIF)

Figure S2 MDP responsiveness in cells transduced with NOD2 virus. Over-expression of NOD2 in HEK293T cells by viral transduction induced secretion of IL-8 and shorter transduction period enhanced responsiveness to MDP. Cells were infected with NOD2 BacMam virus (MOI = 50) for 6 or 24 hours prior to stimulation with MDP (30 ng/mL) for an additional 24 hours. IL-8 secreted into conditioned medium was measured by HTRF assay and is presented as mean ± SD. Data are representative of two independent experiments. (TIF)

Figure S3 The NOD2 inhibitor GSK669 does not block cellular uptake of MDP. Immunofluorescence images of HCT116 cells pre-incubated for 30 minutes with or without GSK669 (10–20 μM) and the endocytosis inhibitor chlorpromazine (20 μM) and then incubated with MDP-Rhodamine (2.5 μg/mL) for 4 hours. (TIF)

Figure S4 Inverse relationship between NOD2 inhibitor concentration and MDP stimulatory activity. (A) Primary human monocytes were pre-treated with one of five different concentrations of GSK717 over the range 0.5–15 μM. For each inhibitor concentration, the cells were then stimulated with a concentration range of MDP (0.03–10 μg/mL) and the amount of IL-8 secreted after 24 hours determined by HTRF assay. The amount of MDP required to obtain a half-maximal IL-8 response (MDP EC_50_) increased as the concentration of inhibitor was increased. (B) Schild plot in which the log[Dose ratio −1] for MDP is plotted against log inhibitor concentration. The dose ratio is defined as the difference in EC_50_ for an agonist between antagonist treated and untreated cells determined at each concentration of antagonist, in this instance EC_50__[MDP alone]_ /EC_50__[MDP with GSK717]_. The linear relationship and gradient of 1.0 demonstrate the apparent competitive behavior between MDP and NOD2 inhibitor. Data are representative of two experiments. (TIF)

Methods S1 Chemical syntheses for all compounds presented in tables (compounds 1–37). (DOC)
