# Supplementary figures and images for: Identification of Benzimidazole Diamides as Selective Inhibitors of the Nucleotide-Binding Oligomerization Domain 2 (NOD2) Signaling Pathway
Source: PLoS One. 2013 Aug 1;8(8):e69619. doi: 10.1371/journal.pone.0069619 (PMC3731320; doi:10.1371/journal.pone.0069619)

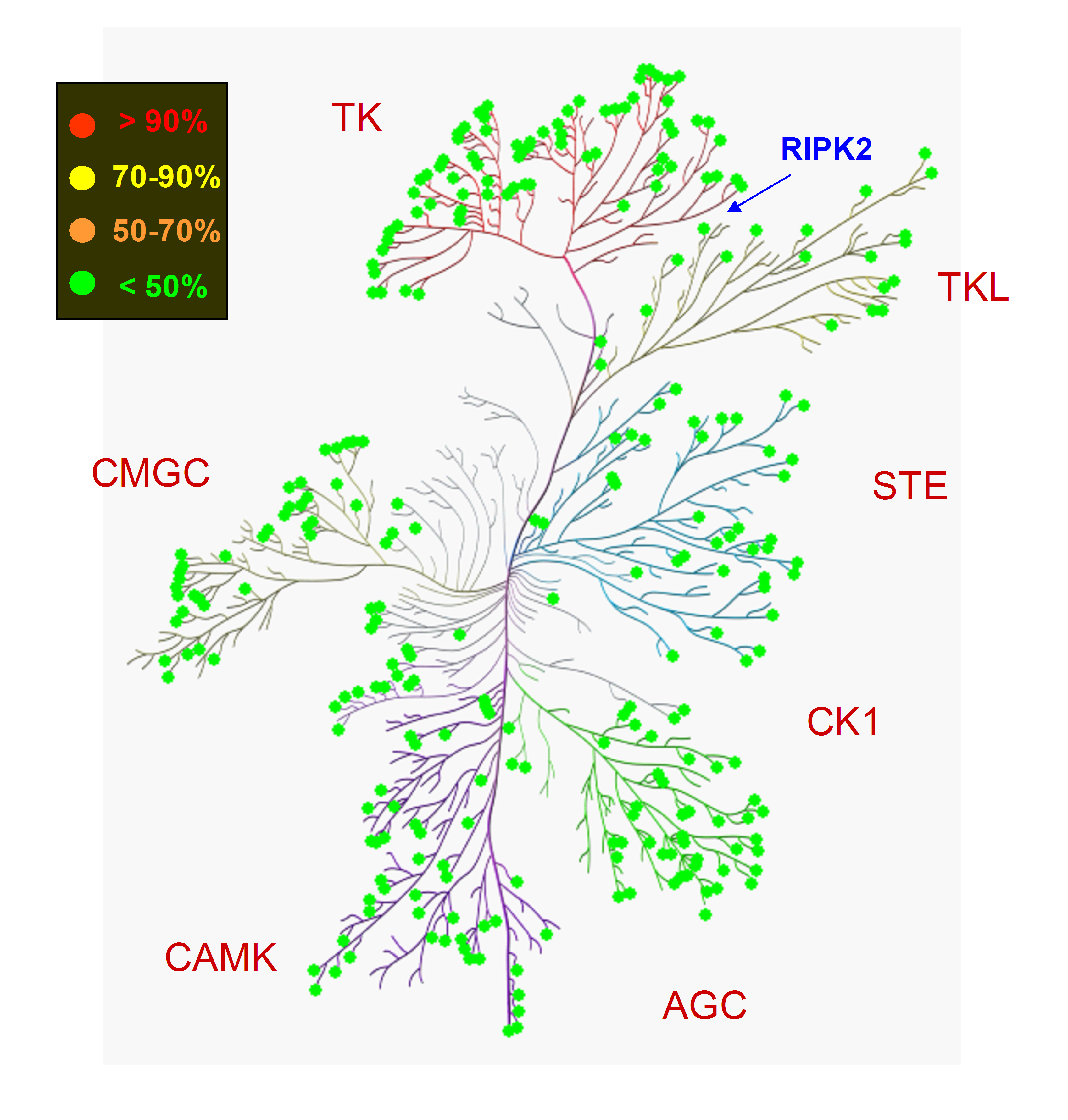

Supplement: Figure S1 — (TIF) [file pone.0069619.s001.tif]

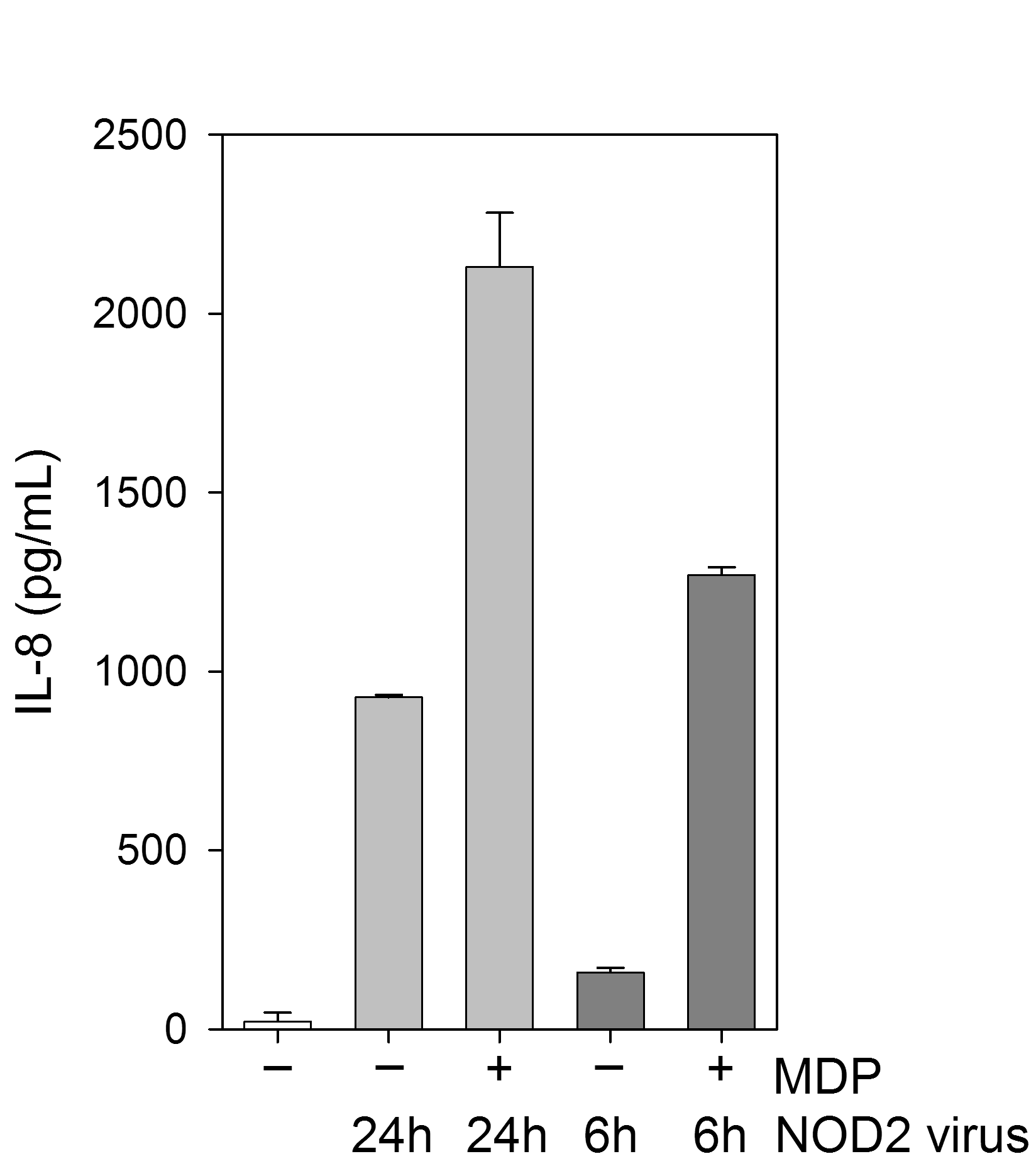

Supplement: Figure S2 — (TIF) [file pone.0069619.s002.tif]

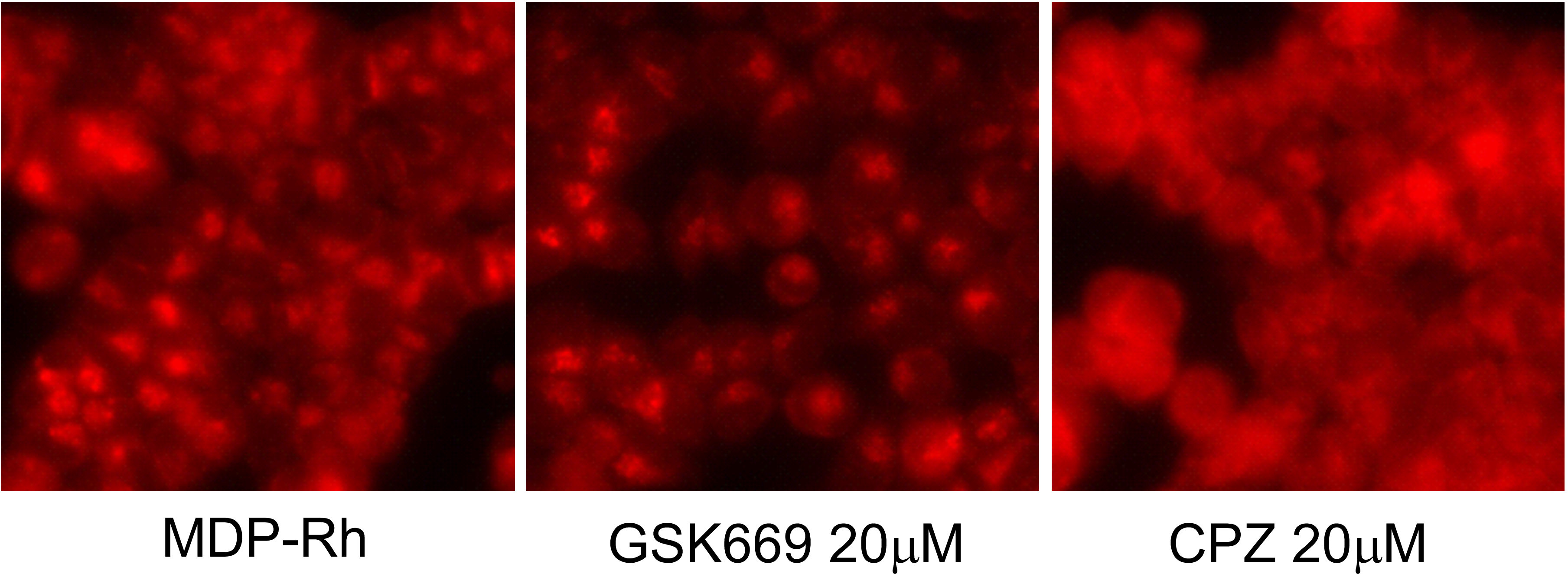

Supplement: Figure S3 — (TIF) [file pone.0069619.s003.tif]

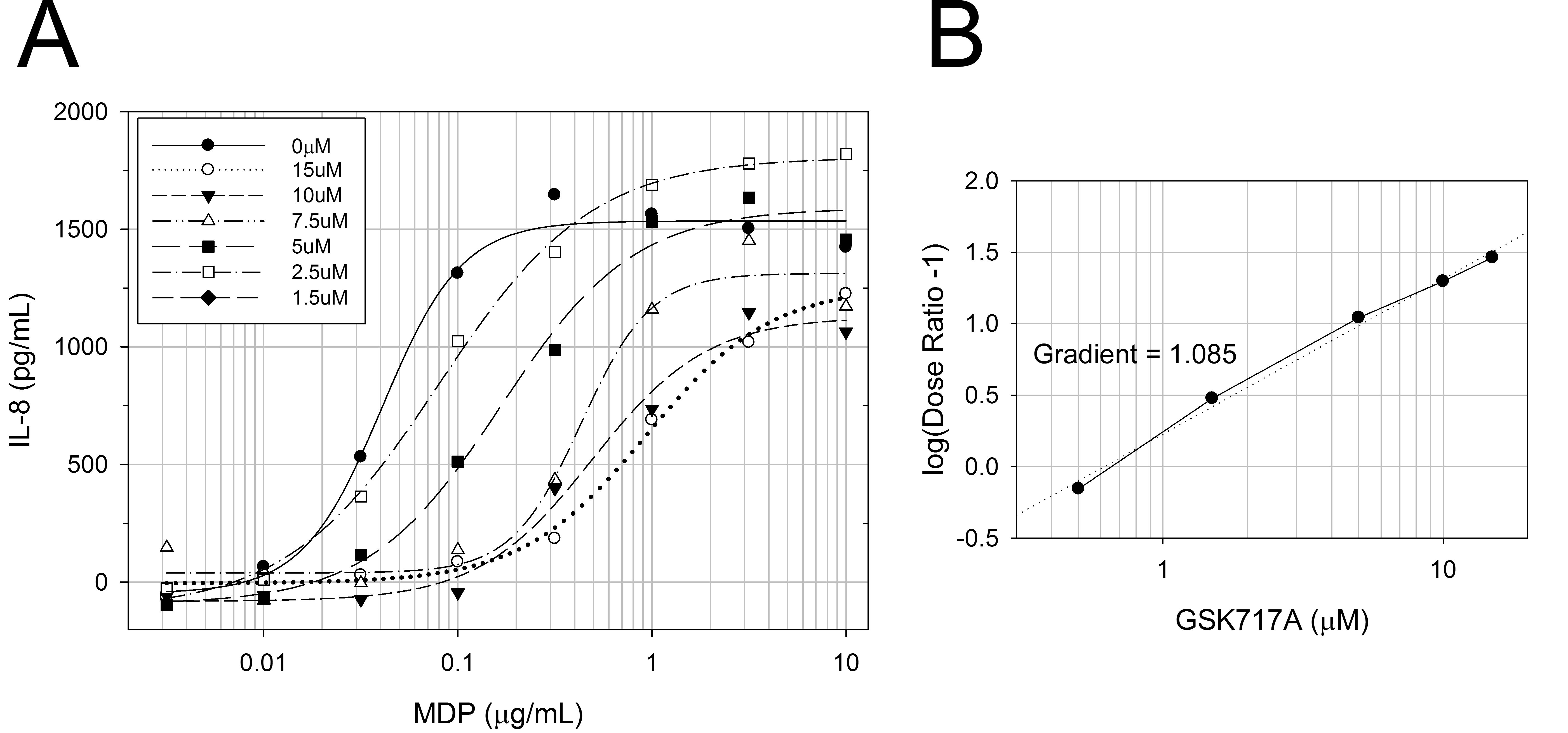

Supplement: Figure S4 — (TIF) [file pone.0069619.s004.tif]
