# Supplementary material for: Identification of Benzimidazole Diamides as Selective Inhibitors of the Nucleotide-Binding Oligomerization Domain 2 (NOD2) Signaling Pathway
Source: PLoS One. 2013 Aug 1;8(8):e69619. doi: 10.1371/journal.pone.0069619 (PMC3731320; doi:10.1371/journal.pone.0069619)
Supplement: Methods S1 — (DOC) [file pone.0069619.s005.doc]

**Supplementary Methods S1**

**Compound syntheses**

Compound **1** N-(2-(1-(2-((2,3-dihydro-1H-inden-5-yl)amino)-2-oxoethyl)-1H-benzo[d]imidazol-2-yl)ethyl)benzamide was a purchased compound and part of the GSK screening collection:

Compound **2** N-(2-(1-(2-((4-isopropylphenyl)amino)-2-oxoethyl)-1H-benzo[d]imidazol-2-yl)ethyl)benzamide was a purchased compound and part of the GSK screening collection:

Synthesis of Compounds **3-14**:

Step-1: Preparation of methyl 2-(2-(2-(tert-butoxycarbonylamino)ethyl)-1H-benzo[d]

imidazol-1-yl)acetate:

To a solution of methyl tert-butyl 2-(1H-benzo[d]imidazol-2-yl)ethylcarbamate (2.0 g, 7.7 mmol) in DMF (20 mL) was added potassium tert-butoxide (1.28 g, 11.5 mmol), stirred for 30 min, then bromomethyl acetate (1.4 g, 9.2 mmol) was added drop wise for 10 min and stirred at rt for 2h. The reaction mixture was diluted with ethyl acetate(50 mL) and washed with water(2 x 10 mL). The organic layer was separated, washed with brine, dried over sodium sulfate, filtered and concentrated to get crude product (LCMS: 46%). The crude product was added to a silica gel (100-200mesh) column and eluted with 5% of MeOH/CHCl3. The product fractions were collected and concentrated to get methyl 2-(2-(2-(tert-butoxycarbonylamino)ethyl)-1H-benzo[d]imidazol-1-yl)acetate (1.7 g, 2.89 mmol, 37 % yield). Compound purity was slightly improved, as such carried over to next step. MS (ES^+^) m/z 334.1 (MH^+^), LCMS purity: 57%.

Step-2: Preparation of methyl 2-(2-(2-aminoethyl)-1H-benzo[d]imidazol-1-yl)acetate:

To a solution of methyl 2-(2-(2-(tert-butoxycarbonylamino)ethyl)-1H-benzo[d]imidazol-1-yl)acetate (1.7 g, 5.1 mmol) in DCM (10 mL) was added 4M HCl in 1,4-dioxane (15 mL, 15 mmol) at 0 oC. The reaction mixture was warmed to rt for 24 h under nitrogen atmosphere, reaction was monitored by TLC. Upon completion the reaction mixture was concentrated under reduced pressure. The residue was obtained was diluted with methanol (40 mL), neutralized the methanol layer with amberlyst A-21 ion exchange resin, and filtered to remove the resin. Thefiltrate was evaporated under reduced pressure to obtain crude methyl 2-(2-(2-aminoethyl)-1H-benzo[d]imidazol-1-yl)acetate (750 mg, 1.8 mmol, 35% yield); as such carried over to next step without purification. MS (ES^+^) m/z 234.3 (MH^+^), LCMS purity: 56%.

Step-3: Preparation of methyl 2-(2-(2-benzamidoethyl)-1H-benzo[d]imidazol-1-yl)

acetate:

To a solution of 2-(2-(2-aminoethyl)-1H-benzo[d]imidazol-1-yl)acetate (750 mg, 3.2 mmol) in Dichloromethane (15 mL) was added benzoic acid (353 mg, 2.89 mmol), EDC. HCl, (927 mg, 4.82 mmol), HOBt (691 mg, 5.12 mmol) and TEA (0.89 mL, 6.4 mmol) at rt. the contents were stirred at rt for 24 h. The reaction was monitored by TLC. Uponcompletion, the reaction mixture was diluted with DCM (15 mL), washed with water (2 × 20 mL), the separated organic layer was washed with brine solution (10 mL), dried over sodium sulfate, filtered and concentrated to afford the 890 mg crude. The crude product was added to a silica gel column (100-200 mesh) and eluted with 0-3% of methanol in DCM. The product fractions collected were concentrated to obtain methyl 2-(2-(2-benzamidoethyl)-1H-benzo[d]imidazol-1-yl)acetate (600 mg, 1.52 mmol, 47 % yield) as an off white powder.

^1^H NMR (DMSO-d6 400 MHz) δ: 3.1-3.18 (t, 2H), 3.73(s, 3H), 4-4.1(q, 2H), 4.88(s, 2H), 7.21-7.31(m, 3H), 7.36-7.43(t, 2H), 7.44-7.5 (d, 1H), 7.68 (b, 1H), 7.74-7.82(m, 3H). MS (ES^+^) m/z 338.1 (MH^+^), LCMS purity: 85 %.

Step-4: Preparation of 2-(2-(2-benzamidoethyl)-1H-benzo[d]imidazol-1-yl)acetic acid:

An aqueous NaOH solution (148 mg, 3.7 mmol, in water 1.5 mL) was added to a methyl 2-(2-(2-benzamidoethyl)-1H-benzo[d]imidazol-1-yl)acetate (500 mg, 1.9 mmol) in THF (10 mL) at 5°C for 2 h and gradually heated to 60°C and stirred for 4 h. The reaction was monitored by TLC (5% MeOH in CHCl3). Upon completion of the reaction, the reaction mixture was cooled to room temperature and evaporated under reduced pressure to remove solvents. Theresidue was diluted with water (10 mL), neutralized with aq. 1N HCl, and extracted with ethyl acetate (5 x 25 mL). Thecombined organics were washed with brine solution, dried over sodium sulfate, filtered and concentrated to afford 2-(2-(2-benzamidoethyl)-1H-benzo[d]imidazol-1-yl)acetic acid (450 mg, 1.39 mmol, 93.9 % yield) as an off white powder.

^1^H NMR (DMSO-d6 400 MHz) δ: 3.05 (t, 3H), 3.72-3.8 (q, 2H), 4.45 (s, 1H), 7.02-7.15 (m, 2H), 7.3-7.4 (m, 3H), 7.4-7.5(m, 2H), 7.88 (d, 2H), 9.05 (b, 1H). MS (ES^+^) m/z 324.1 (MH^+^), LCMS purity: 99.84%.

Compound **3**: Preparation of N-(2-(1-(2-((3-isopropylphenyl)amino)-2-oxoethyl)-1H-benzo

[d]imidazol-2-yl)ethyl)benzamide:

To a solution of 2-(2-(2-benzamidoethyl)-1H-benzo[d]imidazol-1-yl)acetic acid (100 mg, 0.3 mmol) in DMF (2.5 mL) was added 3-isopropylaniline (42mg, 0.32 mmol), EDC. HCl, (88 mg, 0.46 mmol), HOBt (66 mg, 0.48 mmol) and TEA (0.086 mL, 0.62 mmol) at rt. The contents were stirred at rt for 14 h. The reaction mixture was poured on to ice water. A precipitate formed and was filtered. The cake was washed with cold water, ether and then dried under high vacuum to obtain N-(2-(1-(2-(3-isopropylphenylamino)-2-oxoethyl)-1H-benzo[d]imidazol-2-yl)ethyl)benzamide (46 mg, 0.103 mmol, 33% yield) as an white powder. ^1^H NMR (DMSO-d6 400 MHz) δ: 1.15-1.20 (d, 6H), 2.80-2.90 (m, 1H), 3.11-3.20 (t, 2H), 3.70-3.82 (q, 2H), 5.15 (s, 2H), 6.99-7.01 (d, 1H), 7.15-7.25 (m, 3H), 7.35-7.65 (m, 7H), 7.80-7.91 (d, 2H), 8.71 (b, 1H), 10.48 (s, 1H). MS (ES^+^) m/z 441.6 (MH^+^), LCMS purity: 99%.

Compound **4**: Preparation of N-(2-(1-(2-(benzo[b]thiophen-5-ylamino)-2-oxoethyl)-1H-benzo[d]imidazol-2-yl)ethyl)benzamide:

Prepared according to the general procedure above to obtain N-(2-(1-(2-(benzo[b]thiophen-5-ylamino)-2-oxoethyl)-1H-benzo[d]imidazol-2-yl)ethyl)benzamide (38 mg, 0.082 mmol, 26% yield) as an off white powder. ^1^H NMR (DMSO-d6 400 MHz) δ: 3.10-3.20 (t, 2H), 3.80 (q, 2H), 5.18 (s, 2H), 7.20 (m, 2H), 7.36-7.40 (d, 1H), 7.41-7.54 (m, 5H), 7.59-7.64 (d, 1H), 7.74-7.78 (d, 1H), 7.81-7.86 (d, 2H), 7.91-7.96 (d, 1H), 8.21 (b, 1H), 8.60-8.72 (b, 1H), 10.65 (s, 1H). MS (ES^+^) m/z 455.5 (MH^+^), LCMS purity: 98%, HPLC purity: 99%

Compound **5**: Preparation of N-(2-(1-(2-(naphthalen-2-ylamino)-2-oxoethyl)-1H-benzo

[d]imidazol-2-yl)ethyl)benzamide:

Prepared according to the general procedure above to obtain N-(2-(1-(2-(naphthalen-2-ylamino)-2-oxoethyl)-1H-benzo[d]imidazol-2-yl)ethyl)benzamide (56 mg, 0.12 mmol, 38% yield) as an off white powder. ^1^H NMR (DMSO-d6 400 MHz) δ: 3.40-3.60 (b, 3H), 3.80-3.91 (q, 2H), 5.60 (s, 2H), 7.38-7.60 (m, 7H), 7.64-7.77 (m, 1H), 7.74-7.98 (m, 7H), 8.24-8.28 (b, 1H), 8.88 (b, 1H), 11.20 (s, 1H). MS (ES^+^) m/z 449.6 (MH^+^), LCMS purity: 96%.

Compound **6**: Preparation of N-(2-(1-(2-(3,4-dimethylphenylamino)-2-oxoethyl)-1H-benzo

[d]imidazol-2-yl)ethyl)benzamide:

Prepared according to the general procedure above to obtain N-(2-(1-(2-(3,4-dimethylphenylamino)-2-oxoethyl)-1H-benzo[d]imidazol-2-yl)ethyl)benzamide (38 mg, 0.088 mmol, 28% yield) as an white solid. ^1^H NMR (DMSO-d6 400 MHz) δ: 2.1-2.2(s, 6H), 3.08-3.18 (m, 2H), 3.7-3.82(m, 2H), 5.16(s, 2H), 7.06(d, 1H), 7.14-7.22(m, 2H), 7.29 (d, 1H), 7.36-7.38 (b, 1H), 7.42-7.54(m, 4H), 7.58-7.62 (d, 1H), 7.82-7.86 (d, 2H), 10.38 (s, 1H). MS (ES^+^) m/z 427.0 (MH^+^) HPLC purity: 99%.

Compound **7**: Preparation of N-(2-(1-(2-(benzofuran-5-ylamino)-2-oxoethyl)-1H-benzo

[d]imidazol-2-yl)ethyl)benzamide:

Prepared according to the general procedure above to obtain N-(2-(1-(2-(benzofuran-5-ylamino)-2-oxoethyl)-1H-benzo[d]imidazol-2-yl)ethyl)benzamide (85 mg, 0.19 mmol, 61% yield) as a pale brown powder. ^1^H NMR (DMSO-d6 400 MHz) δ: 3.10-3.20 (t, 2H), 3.80 (q, 2H), 5.18 (s, 2H), 6.91 (s, 1H), 7.15-7.25 (m, 2H), 7.40-7.65 (m, 7H), 7.80-7.86 (d, 2H), 7.94-7.98 (d, 2H), 8.65 (b, 1H), 10.55 (s, 1H). MS (ES^+^) m/z 439.5 (MH^+^), LCMS purity: 98%.

Compound **8**: Preparation of N-(2-(1-(2-(3,4-dichlorophenylamino)-2-oxoethyl)-1H-benzo[d]imidazol-2-yl)ethyl)benzamide:

Prepared according to the general procedure above to obtain N-(2-(1-(2-(3,4-dichlorophenylamino)-2-oxoethyl)-1H-benzo[d]imidazol-2-yl)ethyl)benzamide (26 mg, 0.055 mmol, 12% yield) as an pale brown powder. ^1^H NMR (DMSO-d6 400 MHz) δ: 3.08-3.18 (m, 2H), 3.7-3.82(m, 2H), 5.16(s, 2H), 7.15-7.22(t, 2H), 7.4-7.55(m, 4H), 7.55-7.65 (m, 2H), 7.8-7.88 (d, 2H), 7.95(b, 1H), 8.68 (b, 1H), 10.8 (s, 1H). MS (ES^+^) m/z 467.5 (MH^+^), LCMS purity: 98.81%.

Compound **9**: Preparation of N-(2-(1-(2-((3-hydroxy-2,3-dihydro-1H-inden-5-yl)amino)-2-oxoethyl)-1H-benzo[d]imidazol-2-yl)ethyl)benzamide:

Prepared according to the general procedure above to obtain N-(2-(1-(2-(3-hydroxy-2,3-dihydro-1H-inden-5-ylamino)-2-oxoethyl)-1H-benzo[d]imidazol-2-yl)ethyl)

benzamide (93 mg, 0.19 mmol, 60% yield) as an off white powder. ^1^H NMR (DMSO-d6 400 MHz) δ: 1.68-1.80 (m, 1H), 2.45-2.80 (b, 1H), 2.60-2,70 (m, 1H), 2.75-2.90 (b, 1H), 3.10-3.20 (t, 2H), 3.71-3.85 (m, 2H), 4.90-5.02 (m, 1H), 5.15 (s, 2H), 5.22 (d, 1H), 7.12-7.23 (m, 3H), 7.35-7.40 (d, 1H), 7.42-7.55 (m, 4H), 7.56-7.62 (b, 2H), 7.80-7.90 (d, 2H), 8.70 (b, 1H), 10.45 (s, 1H). MS (ES^+^) m/z 455.5 (MH^+^) LCMS purity: 91%.

Compound **10**: Preparation of N-(2-(1-(2-(indolin-5-ylamino)-2-oxoethyl)-1H-benzo[d]imidazol-2-yl)ethyl)benzamide:

Prepared according to the general procedure above to obtain N-(2-(1-(2-(indolin-5-ylamino)-2-oxoethyl)-1H-benzo[d]imidazol-2-yl)ethyl)benzamide (80 mg, 0.16 mmol, 25% yield) as a brown powder. ^1^H NMR (DMSO-d6 400 MHz) δ: 2.8-2.9 (t, 2H), 3.0-3.1 (t, 2H), 3.1-3.2(m, 4H), 3.7-3.8(m, 4H), 4.6(t, 2H), 4.9(s, 2H), 5.07(s, 2H), 5.28(s, 2H), 5.32(b, 1H), 6.3(d, 1H), 6.41(d, 1H), 6.5(s, 1H), 7.08(d, 1H), 7.14-7.22(m, 4H), 7.28(b, 1H), 7.4-7.54(m, 8H), 7.58-7.62(t, 3H), 7.8-7.9(d, 4H), 8.62-8.72(m, 2H), 10.12(s, 1H). MS (ES^+^) m/z 440.3 (MH^+^), LCMS purity: 87%.

Compound **11**: Preparation of N-(2-(1-(2-((4-(benzyloxy)phenyl)amino)-2-oxoethyl)-1H-benzo[d]imidazol-2-yl)ethyl)benzamide:

Prepared according to the general procedure above to obtain N-(2-(1-(2-(4-(benzyloxy)phenylamino)-2-oxoethyl)-1H-benzo[d]imidazol-2-yl)ethyl)benzamide (98 mg, 0.19 mmol, 62% yield) as an off white powder. ^1^H NMR (DMSO-d6 400 MHz) δ: 3.11-3.18 (t, 2H), 3.70-3.82 (q, 2H), 5.10 (s, 2H), 5.13 (s, 2H), 6.94-6.98 (d, 2H), 7.18 (m, 2H), 7.28-7.54 (m, 11H), 7.58-7.62 (m, 2H), 7.82-7.86 (d, 2H), 8.65 (b, 1H), 10.38 (s, 1H). MS (ES^+^) m/z 505.5 (MH^+^), LCMS purity: 99%, HPLC purity: 98%.

Compound **12**: Preparation of N-(2-(1-(2-(indolin-6-ylamino)-2-oxoethyl)-1H-benzo[d]imidazol-2-yl)ethyl)benzamide:

Prepared according to the general procedure above to obtain N-(2-(1-(2-(indolin-6-ylamino)-2-oxoethyl)-1H-benzo[d]imidazol-2-yl)ethyl)benzamide (45 mg, 0.083 mmol, 27% yield) as a brown powder. ^1^H NMR (DMSO-d6 400 MHz) δ: 2.8-2.9 (t, 2H), 3.1-3.18 (t, 2H), 3.38-3.41(t, 2H), 3.7-3.82 (b, 2H), 5.1(st, 2H), 5.55 (s, 1H), 6.68 (d, 1H), 6.84-6.94 (m, 2H), 7.14-7.22 (m, 2H), 7.40-7.62 (m, 5H), 7.82-7.84 (d, 2H), 8.60-8.70 (b, 1H), 10.2 (s, 1H). MS (ES^+^) m/z 440.4 (MH^+^), LCMS purity: 81%.

Compound **13**: Preparation of N-(2-(1-(2-oxo-2-((4-phenoxyphenyl)amino)ethyl)-1H-benzo

[d]imidazol-2-yl)ethyl)benzamide:

Prepared according to the general procedure above to obtain N-(2-(1-(2-oxo-2-(4-phenoxyphenylamino)ethyl)-1H-benzo[d]imidazol-2-yl)ethyl)benzamide (81 mg, 0.17 mmol, 53.2% yield) as a white powder. ^1^H NMR (DMSO-d6 400 MHz) δ: 3.11-3.20 (t, 2H), 3.75-3.82 (q, 2H), 5.15 (s, 2H), 6.94-7.02 (m, 4H), 7.08-7.12 (t, 1H), 7.15-7.24 (m, 2H), 7.32-7.38 (t, 2H), 7.42-7.54 (m, 4H), 7.56-7.64 (d, 3H), 7.82-7.86 (d, 2H), 8.71 (b, 1H), 10.55 (s, 1H). MS (ES^+^) m/z 491.0 (MH^+^), LCMS purity: 99%, HPLC purity: 99%

Compound **14**: Preparation of N-(2-(1-(2-(1H-indol-5-ylamino)-2-oxoethyl)-1H-benzo[d]

imidazol-2-yl)ethyl)benzamide:

Prepared according to the general procedure above to obtain N-(2-(1-(2-(1H-indol-5-ylamino)-2-oxoethyl)-1H-benzo[d]imidazol-2-yl)ethyl)benzamide (110 mg, 0.25 mmol, 54% yield) as an pale brown powder. ^1^H NMR (DMSO-d6 400 MHz) δ: 3.12-3.2 (t, 2H), 3.72-3.80 (m, 2H), 5.15(s, 2H), 6.35 (s, 1H), 7.15-7.25 (m, 3H), 7.28-7.35. (m, 2H), 7.40-7.55 (m, 4H), 7.6 (d, 1H),7.80-7.90 (b, 3H), 8.7 (b, 1H), 10.32 (s, 1H), 11.01 (s, 1H). MS (ES^+^) m/z 438.6 (MH^+^), LCMS purity: 98%.

Synthesis of Compounds **15-22**:

Step-1: Preparation of tert-butyl (3-((2-aminophenyl)amino)-3-oxopropyl)carbamate:

To a stirred solution of N-Boc – β alanine (50g, 260 mmol) in THF (1000 mL) were added EDC^.^HCl (76 g, 400 mmol), HOBt (60 g, 400 mmol) and 1,2-diamino benzene (34 g, 290 mmol). To this mixture was added TEA (56 mL, 400 mmol) dropwise over a period of 20 min at rt. The reaction mixture was stirred for overnight at rt and then quenched with water (250 mL) and concentrated under reduced pressure to remove THF; the resulting residue was dissolved in DCM (2000 mL), washed with water (2 x 500 mL), dried over anhydrous Na_2_SO_4_, filtered and concentrated to afford the title compound (56.8 g, 204 mmol, 44% crude yield), which was taken to the next step without any purification. MS (ES^-^) m/z 278.5 (MH^-^), LCMS purity: 67%.

Step-2: Preparation of tert-butyl (2-(1H-benzo[d]imidazol-2-yl)ethyl)carbamate:

A solution of tert-butyl (3-((2-aminophenyl)amino)-3-oxopropyl)carbamate (25 g, 90 mmol) in AcOH (250 mL) was heated at 65 °C for 1 hr. The reaction mixture was concentrated under reduced pressure to remove AcOH. The resulting residue was dissolved in DCM (1000 mL) andwashed with saturatedNaHCO_3_ solution (3 x 150 mL) and water (100 mL), The organic layer was separated and dried over anhydrous Na_2_SO_4_, filtered and concentrated to afford the title compound as crude (26.3 g, 101 mmol, quantitative yield), which was taken to the next step without any purification.

Step-3: Preparation of methyl 2-(2-(2-((tert-butoxycarbonyl)amino)ethyl)-1H-benzo[d]

imidazol-1-yl)acetate:

To a stirred solution of tert-butyl (2-(1H-benzo[d]imidazol-2-yl)ethyl)carbamate (26 g, 100 mmol) in DMF (400 mL) were added KOt-Bu (16.7 g, 150 mmol) followed by Methylbromoacetate (11 mL, 120 mmol) at 0 °C. The reaction mixture was allowed to stir for 1 hr at rt and quenched with water (500 mL). The mixture was extracted with diethyl ether (2 x 200 mL), dried over anhydrous Na_2_SO_4_, filtered and concentrated to afford the title compound as crude (32.5 g, 97.6 mmol, 98% yield), which was taken to the next step without any purification.

Step-4: Preparation of 2-(2-(2-((tert-butoxycarbonyl)amino)ethyl)-1H-benzo[d]imidazol-1-yl)acetic acid:

A solution of methyl 2-(2-(2-((tert-butoxycarbonyl)amino)ethyl)-1H-benzo[d]imidazol-1-yl)acetate (8 g, 24 mmol) in THF (50 mL) was added LiOH^.^H2O (1.5 g, 36 mmol) in H_2_O (10 mL) and the resulting solution was stirred for overnight at rt. The reaction mixture was concentrated under reduced pressure to dryness and the resulting solid (10 g, 31 mmol, quantitative yield) was taken to the next step without any purification.

Step-5: Preparation of tert-butyl (2-(1-(2-((2,3-dihydro-1H-inden-5-yl)amino)-2-oxoethyl)-1H-benzo[d]imidazol-2-yl)ethyl)carbamate:

To a stirred solution of 2-(2-(2-((tert-butoxycarbonyl)amino)ethyl)-1H-benzo[d]imidazol-1-yl)acetic acid (10g, 31 mmol) in DMF (200 mL) were added 5-amino indane (4.16 g, 31.3 mmol), EDC^.^HCl (9.0 g, 47 mmol), HOBt (6.3 g, 47 mmol) and TEA (6.6 mL, 47 mmol) drop wise over a period of 20 min at rt. The reaction mixture was stirred overnight at rt. TLC indicated completion of the reaction. The reaction mixture was quenched with water (200 mL), extracted with ethyl acetate (2 x 100 mL), dried over anhydrous Na_2_SO_4_, filtered and concentrated to afford the title compound as crude (3.6 g, 8.3 mmol, 26% crude yield); which was taken to the next step without any purification. MS (ES^+^) m/z 435.6 (MH^+^), HPLC purity: 83%.

Step-6: Preparation of 2-(2-(2-aminoethyl)-1H-benzo[d]imidazol-1-yl)-N-(2,3-dihydro-1H-inden-5-yl)acetamide :

A solution of tert-butyl(2-(1-(2-((2,3-dihydro-1H-inden-5-yl)amino)-2-oxoethyl)-1H-benzo[d]imidazol-2-yl)ethyl)carbamate (3.6 g, 8.3 mmol) in DCM (20 mL) was added TFA (20 mL) at 0 °C and the resulting mixture was stirred for 30 min at rt. TLC indicated completion of the reaction. The reaction mixture was concentrated under reduced pressure, and the resulting residue was dissolved in water (30 mL) and extracted with DCM (2 x 50 mL). The organic layer was separated and discarded. The pH of the aqueous layer was adjusted to pH = 10 using 6N Na_2_CO_3_ solution. The aqueous layer was extracted with DCM (2 x 100 mL). The organic layer was separated and dried over anhydrous Na_2_SO_4_, filtrated and concentrated to afford (1.65 g, 4.94 mmol, 59% yield) of the title compound as a pale yellow powder. MS (ES^-^) m/z 333.5 (MH^-^), LCMS purity: 81%.

Compound **15**: Preparation of N-(2-(1-(2-((2,3-dihydro-1H-inden-5-yl)amino)-2-oxoethyl)-1H-benzo[d]imidazol-2-yl)ethyl)-1-naphthamide.

To a stirred solution of 2-(2-(2-aminoethyl)-1H-benzo[d]imidazol-1-yl)-N-(2,3-dihydro-1H-inden-5-yl)acetamide (90 mg, 0.269 mmol) in dry DMF (5 mL) were added EDC^.^HCl (77 mg, 0.40 mmol), HOBt (54 mg, 0.40 mmol), 1-Naphthoic acid (46 mg, 0.27 mmol) and TEA (0.056 mL, 0.40 mmol) at rt. The reaction mixture was stirred for overnight at rt. TLC indicated completion of the reaction. The reaction mixture was diluted with water (100 mL) and the resultant solid was filtered and dried to afford the title compound (65 mg, 0.133mmol, 49% yield) as a brown color powder. ^1^H NMR (DMSO-d_6_, 400 MHz) δ: 1.95-2.10 (m, 2H), 2.8-2.85 (q, 4H), 3.20-3.25 (m, 2H), 3.80-3.85 (q, 2H), 5.20 (s, 2H), 7.12-7.20 (m, 3H),7.25-7.30 (d, 1H), 7.40-7.55 (m, 5H), 7.58-7.61 (t, 2H), 7.70-7.81 (q, 2H), 8.10-8.15 (d, 1H), 8.60-8.70 (t, 1H), 10.40 (s, 1H). MS (ES^+^) m/z 489.0 (MH^+^), HPLC purity: 97%.

Compound **16**: Preparation of 2-chloro-N-(2-(1-(2-((2,3-dihydro-1H-inden-5-yl)amino)-2-oxoethyl)-1H-benzo[d]imidazol-2-yl)ethyl)benzamide.

Prepared according to the general procedure above to obtain 2-chloro-N-(2-(1-(2-((2,3-dihydro-1H-inden-5-yl)amino)-2-oxoethyl)-1H-benzo[d]imidazol-2-yl)ethyl)benzamide (48 mg, 0.101 mmol, 37% yield). ^1^H NMR (DMSO-d_6_, 400 MHz) δ: 1.95-2.1 (m, 2H), 2.8-2.85 (q, 4H), 3.1-3.25 (m, 2H), 3.75-3.8 (q, 2H), 5.20-5.30 (s, 2H), 7.12-7.18 (d, 1H),7.25-7.30 (m, 1H), 7.30-7.35 (m, 1H), 7.36-7.40 (m, 1H), 7.40-7.46 (m, 3H), 7.48-7.52 (m, 2H), 7.60-7.64 (m, 1H), 7.68-7.72 (m, 1H), 8.60-8.70 (t, 1H), 10.40-10.50 (s, 1H). MS (ES^+^) m/z 473.0 (MH^+^), HPLC purity: 95%.

Compound **17**: Preparation of N-(2-(1-(2-((2,3-dihydro-1H-inden-5-yl)amino)-2-oxoethyl)-1H-benzo[d]imidazol-2-yl)ethyl)-2-methylbenzamide.

Prepared according to the general procedure above to obtain N-(2-(1-(2-((2,3-dihydro-1H-inden-5-yl)amino)-2-oxoethyl)-1H-benzo[d]imidazol-2-yl)ethyl)-2-methylbenzamide (31 mg, 0.068 mmol, 25% yield). ^1^H NMR (DMSO-d_6_, 400 MHz) δ: 2.0-2.1 (m, 2H), 2.20 (s, 3H), 2.80 (t, 4H), 3.20 (t, 2H), 3.6-3.80 (t, 2 H) 5.1-5.15 (s, 2H), 7.1-7.40 (m, 8H), 7.40-7.45 (s, 1H), 7.6-7.7 (m, 2H), 8.4-8.43 (t, 1H), 10.4-10.43 (s, 1H). MS (ES^+^) m/z 453.3 (MH^+^), LCMS purity: 95%.

Compound **18**: Preparation of N-(2-(1-(2-((2,3-dihydro-1H-inden-5-yl)amino)-2-oxoethyl)-1H-benzo[d]imidazol-2-yl)ethyl)-4-methylbenzamide.

Prepared according to the general procedure above to obtain N-(2-(1-(2-((2,3-dihydro-1H-inden-5-yl)amino)-2-oxoethyl)-1H-benzo[d]imidazol-2-yl)ethyl)-4-methylbenzamide (57 mg, 0.126 mmol, 47% yield). ^1^H NMR (DMSO-d_6_, 400 MHz) δ: 1.9-2.1 (m, 2H), 2.30-2.35 (s, 3H), 2.75-2.85 (q, 4H), 3.10-3.20 (t, 2H), 3.70-3.80 (q, 2 H) 5.1-5.15 (s, 2H), 7.10-7.30 (m, 6H), 7.40-7.50 (m, 2H), 7.60-7.63 (d, 1H), 7.70-7.80 (d, 2H), 8.55-8.62 (t, 1H), 10.4-10.43 (s, 1H). MS (ES^-^) m/z 451.1 (MH^-^), HPLC purity: 96%.

Compound **19**: Preparation of 3,4-dichloro-N-(2-(1-(2-((2,3-dihydro-1H-inden-5-yl)amino)-2-oxoethyl)-1H-benzo[d]imidazol-2-yl)ethyl)benzamide.

Prepared according to the general procedure above to obtain 3,4-dichloro-N-(2-(1-(2-((2,3-dihydro-1H-inden-5-yl)amino)-2-oxoethyl)-1H-benzo[d]imidazol-2-yl)ethyl)benzamide (40 mg, 0.079 mmol, 35% yield). ^1^H NMR (DMSO-d_6_, 400 MHz) δ: 2.0-2.1 (m, 2H), 2.7-2.8 (t, 4H), 2.90-3.0 (t, 2H), 3.7-3.8 (q, 2H), 5.1-5.15 (s, 2H), 7.1-7.25 (m, 4H), 7.45-7.35 (d, 2H), 7.57-7.61 (m, 1H), 7.7-7.75 (d, 1H), 7.7- 7.81 (m, 1 H), 8.0- 8.05 (d, 1 H), 8.8-8.85 (t, 1H), 10.3-10.4 (s, 1H). MS (ES^+^) m/z 507.0 (MH^+^), HPLC purity: 97%.

Compound **20**: Preparation of N-(2-(1-(2-((2,3-dihydro-1H-inden-5-yl)amino)-2-oxoethyl)-1H-benzo[d]imidazol-2-yl)ethyl)-3-(trifluoromethyl)benzamide.

Prepared according to the general procedure above to obtain N-(2-(1-(2-((2,3-dihydro-1H-inden-5-yl)amino)-2-oxoethyl)-1H-benzo[d]imidazol-2-yl)ethyl)-3-(trifluoromethyl)benzamide (50 mg, 0.098 mmol, 43% yield). ^1^H NMR (DMSO-d_6_, 400 MHz) δ: 2.0-2.1 (m, 2H), 2.7-2.8 (t, 4H), 2.90-3.0 (t, 2H), 3.7-3.8 (q, 2H), 5.1-5.15 (s, 2H), 7.1-7.25 (m, 3H),7.25-7.30 (d, 1H), 7.45-7.35 (d, 2H), 7.57-7.61 (m, 1H), 7.65-7.75 (t, 1H), 7.87- 7.92 (d, 1 H), 8.1- 8.2 (t, 2 H), 8.95-9.0 (t, 1H), 10.3-10.4 (s, 1H). MS (ES^+^) m/z 507.0 (MH^+^), HPLC purity: 90%.

Compound **21**: Preparation of N-(2-(1-(2-((2,3-dihydro-1H-inden-5-yl)amino)-2-oxoethyl)-1H-benzo[d]imidazol-2-yl)ethyl)-3-methylbenzamide.

Prepared according to the general procedure above to obtain N-(2-(1-(2-((2,3-dihydro-1H-inden-5-yl)amino)-2-oxoethyl)-1H-benzo[d]imidazol-2-yl)ethyl)-3-methylbenzamide (50 mg, 0.110 mmol, 48% yield). ^1^H NMR (DMSO-d_6_, 400 MHz) δ: 1.95-2.1 (m, 2H), 2.8-2.85 (q, 4H), 3.1-3.2 (t, 2H), 3.75-3.8 (q, 2H), 5.1-5.15 (s, 2H), 7.1-7.25 (m, 3H),7.25-7.35 (m, 3H), 7.42-7.5 (m, 2H), 7.59-7.69 (m, 3H), 8.6-8.67 (t, 1H), 10.40 (s, 1H). MS (ES^+^) m/z 453.1 (MH^+^), HPLC purity: 97%.

Compound **22**: Preparation of 3-chloro-N-(2-(1-(2-((2,3-dihydro-1H-inden-5-yl)amino)-2-oxoethyl)-1H-benzo[d]imidazol-2-yl)ethyl)benzamide.

Prepared according to the general procedure above to obtain 3-chloro-N-(2-(1-(2-((2,3-dihydro-1H-inden-5-yl)amino)-2-oxoethyl)-1H-benzo[d]imidazol-2-yl)ethyl)benzamide (45 mg, 0.095 mmol, 42% yield). ^1^H NMR (DMSO-d_6_, 400 MHz) δ: 1.95-2.1 (m, 2H), 2.8-2.85 (q, 4H), 3.1-3.2 (t, 2H), 3.75-3.8 (q, 2H), 5.1-5.15 (s, 2H), 7.1-7.25 (m, 3H),7.25-7.35 (d, 1H), 7.4-7.5 (m, 3H), 7.6-7.65 (m, 2H), 7.78-7.80 (d, 1H), 7.85-7.80 (s, 1H), 8.8-8.88 (t, 1H), 10.35-10.40 (s, 1H). MS (ES^+^) m/z 473.0 (MH^+^), HPLC purity: 98%.

Synthesis of Compound **23**:

Step 1: Preparation of methyl 2-(2-(2-aminoethyl)-5-chloro-1H-benzo[d]imidazol-1-yl) acetate:

To a solution of methyl 2-(2-(2-((tert-butoxycarbonyl)amino)ethyl)-5-chloro-1H-benzo[d]imidazol-1-yl)acetate (500 mg, 1.4 mmol) in dichloromethane (DCM) (5mL) was added TFA (1.0 mL, 13 mmol) at 0 ºC and then allowed to rt for 2h. The solvent was removed under reduced pressure and the resulting residue was dissolved in methanol (20 mL), basified with amberlyst A-21 ion exchange resin up to pH~8, the resin filtered, and filtrate was concentrated to obtain methyl 2-(2-(2-aminoethyl)-5-chloro-1H-benzo[d]imidazol-1-yl)acetate (300 mg, 1.0 mmol, 76 % yield) as an orange gum, which was carried over to the next step without any further purification. MS (ES^+^) m/z 268 (MH^+^), LCMS purity 92%

Step 2: Preparation of methyl 2-(2-(2-benzamidoethyl)-5-chloro-1H-benzo[d]imidazol-1-yl) acetate:

To a solution of benzoic acid (164 mg, 1.345 mmol) in dichloromethane (DCM) (10 mL) was added EDC (322 mg, 1.68 mmol), HOBt (257 mg, 1.68 mmol), and TEA (0.234 mL, 1.68 mmol) at rt and stirred for 30 min. Methyl 2-(2-(2-aminoethyl)-5-chloro-1H-benzo[d]imidazol-1-yl)acetate (300 mg, 1.121 mmol) was then added. The resulting mixture was stirred at rt for 16h, the reaction was monitored by TLC (R_f_: 0.45, 5% MeOH in DCM). Upon completion, the reaction was diluted with water (50 mL) and extracted with EtOAc (2 x 50 mL). The combined organics were dried over anhydrous Na_2_SO^4^, filtered and concentrated to obtain methyl 2-(2-(2-benzamidoethyl)-5-chloro-1H-benzo[d]imidazol-1-yl)acetate (320 mg, 0.643 mmol, 57% yield) as colorless oil, which was carried over to the next step without any further purification. MS (ES^+^) m/z 372 (MH^+^), LCMS purity 74%

Step 3: Preparation of 2-(2-(2-benzamidoethyl)-5-chloro-1H-benzo[d]imidazol-1-yl) acetic acid:

1N NaOH (5 eq) was added to a solution of methyl 2-(2-(2-benzamidoethyl)-5-chloro-1H-benzo[d]imidazol-1-yl)acetate (320 mg, 0.861 mmol) in tetrahydrofuran (THF) (10 mL) and water (10.00 mL) at rt for 3 h. The reaction was monitored by TLC (R_f_:0.1, 20% MeOH in DCM). Upon completion, the reaction was diluted with water (10 mL) and extracted with ethyl acetate (to remove the impurities). The aqueous layer was separated and acidified with aq. 5N HCl solution (pH~2), then extracted with 10% MeOH in DCM (5 x 25 mL). The combined organics were dried over anhydrous Na_2_SO_4_, filtered and concentrated to obtain 2-(2-(2-benzamidoethyl)-5-chloro-1H-benzo[d]imidazol-1-yl)acetic acid (160 mg, 0.353 mmol, 41% yield) as colorless oil. MS (ES^+^) m/z 358 (MH^+^), LCMS purity 78%.

Compound **23**: Preparation of N-(2-(5-chloro-1-(2-((2,3-dihydro-1H-inden-5-yl)amino)-2-oxoethyl) -1H-benzo[d]imidazol-2-yl)ethyl)benzamide:

To a solution of 2-(2-(2-benzamidoethyl)-5-chloro-1H-benzo[d]imidazol-1-yl)acetic acid (160 mg, 0.44 mmol) in N,N-Dimethylformamide (DMF) (10 mL) was added EDC (129 mg, 0.671 mmol), HOBt (103 mg, 0.671 mmol) and TEA (0.093 mL, 0.67 mmol) at rt and stirred for 30 min. 2,3-Dihydro-1H-inden-5-amine (71.5 mg, 0.537 mmol) was then added. The resulting mixture was stirred at rt for 16h. The reaction was monitored by TLC (R_f_: 0.3, 5% MeOH in DCM). Uponcompletion, the reaction diluted with water (50 mL), extracted with EtOAc (2 x 50 mL), and the combined organics were dried over anhydrous Na_2_SO_4_, filtered and concentrated to obtain the crude mixture, which was triturated with diethyl ether and dried under vacuum to afford N-(2-(5-chloro-1-(2-((2,3-dihydro-1H-inden-5-yl)amino)-2-oxoethyl)-1H-benzo[d]imidazol-2-yl)ethyl)benzamide (150 mg, 0.32 mmol, 70% yield) as an off white solid. ^1^H NMR (DMSO-d_6_, 400 MHz): δ 1.98-2.02 (t, 2H), 2.75-2.85 (m, 4H), 2.95-3.05 (m, 1H), 3.16 (t, 2H), 3.75-3.79 (t, 2H), 5.13 (s, 2H), 7.12-7.16 (d, 1H), 7.22-7.29 (m, 2H), 7.42-7.53 (m, 5H), 7.66-7.83 (b, 1H), 7.82-7.85 (d, 2H), 8.66 (m, 1H), 10.41 (s, 1H). MS (ES^+^) m/z 473 (MH^+^), LCMS purity 99%.

Synthesis of Compound **24**:

Step 1: Preparation of tert-butyl(3-((2-amino-4-chlorophenyl)amino)-3-oxopropyl) carbamate with tert-butyl (3-((2-amino-5-chlorophenyl)amino)-3-oxopropyl)carbamate:

To a solution of 4-chlorobenzene-1,2-diamine (2.0 g, 14 mmol) in THF (25 mL) was added 3-((tert-butoxycarbonyl)amino)propanoic acid (2.65 g, 14.0 mmol), EDC (4.01 g, 21.0 mmol), HOBt (2.84 g, 21.0 mmol) and TEA (2.91 mL, 21.0 mmol) at rt. The resulting mixture was stirred at rt for 16 h. The reaction mixture was diluted with water (75 mL) and extracted with EtOAc (2 x 75 mL). The combined organics were dried over anhydrous Na_2_SO_4_, filtered and concentrated to obtain the crude mixture, which was purified by silica gel (100-200 mesh) column chromatography by using 50% of EtOAc in pet ether as an eluent to afford tert-butyl (3-((2-amino-4-chlorophenyl)amino)-3-oxopropyl)carbamate compound with tert-butyl (3-((2-amino-5-chlorophenyl)amino)-3-oxopropyl)carbamate (1:1) as pale brown solid (2.0 g, 45% yield). MS (ES^+^) m/z 314 (MH^+^), LCMS purity 81%.

Step 2: Preparation of (1: 1) tautomeric mixture tert-butyl (2-(5-chloro-1H-benzo[d]imidazol-2-yl)ethyl) carbamate & tert-butyl (2-(6-chloro-1H-benzo[d]imidazol-2-yl)ethyl) carbamate:

*t*-Butyl (3-((2-amino-4-chlorophenyl)amino)-3-oxopropyl)carbamate with tert-butyl (3-((2-amino-5-chlorophenyl)amino)-3-oxopropyl)carbamate (1:1) (2.0 g, 3.2 mmol) was dissolved in acetic acid (25 mL) and heated at 70 °C for 3 h, then cooled down to rt, and concentrated under reduced pressure. The residue was dissolved in DCM (50 mL) and washed with aqueous sat. NaHCO_3_The organics were separated and dried over anhydrous Na_2_SO_4_, filtered and concentrated to obtain tert-butyl (2-(5-chloro-1H-benzo[d]imidazol-2-yl)ethyl)carbamate compound with tert-butyl (2-(6-chloro-1H-benzo[d]imidazol-2-yl)ethyl)carbamate (1:1) (1.7g, 2.3 mmol, 73% yield) as an orange gum, which was carried over to the next step without any further purification. MS (ES^+^) m/z 296 (MH^+^), LCMS purity 81%.

Step 3: Preparation of regiomeric mixture of methyl 2-(2-(2-((tert-butoxycarbonyl)amino)ethyl)-6-chloro-1H-benzo[d]imidazol-1-yl)acetate & methyl 2-(2-(2-((tert-butoxycarbonyl)amino)ethyl)-5-chloro-1H-benzo[d]imidazol-1-yl)acetate:

To a solution of tert-butyl (2-(5-chloro-1H-benzo[d]imidazol-2-yl)ethyl)carbamate with tert-butyl (2-(6-chloro-1H-benzo[d]imidazol-2-yl)ethyl)carbamate (1:1) (1.7g, 2.87 mmol) in N,N-dimethylformamide (DMF) (30 mL) was added KOtBu (0.484 g, 4.31 mmol) and methyl bromoacetate (0.318 mL, 3.45 mmol) at 0 ºC under nitrogen atmosphere. The resulting mixture was stirred at rt for 3 hr. the reaction mixture was diluted with water (100mL), extracted with EtOAc (2 x 50 mL), and the combined organics were dried over anhydrous Na_2_SO_4_, filtered and concentrated to obtain a crude mixture, which was purified by silica gel (100-200 mesh) column chromatography by using 60% EtOAC in pet ether as an eluent to afford the desired mixture as an off white solid (1.3 g, regiomers), which was purified on preparative HPLC by using Hexane: ethanol (7:3) as an eluent to afford methyl 2-(2-(2-((tert-butoxycarbonyl)amino)ethyl)-5-chloro-1H-benzo[d]imidazol-1-yl)acetate (500 mg, 1.2 mmol, 42% yield) and methyl 2-(2-(2-((tert-butoxycarbonyl)amino)ethyl)-6-chloro-1H-benzo[d]imidazol-1-yl)acetate (500 mg, 1.3 mmol, 44.0 % yield) as an off white solids. These products were confirmed by NOESY. Following conditions were used to separate the regioisomers. Column: Chiral pak IC (4.6 x 250mm) 5μ, Mobile phase: A: Hexane, B: EtOH, ISO: A: B (70:30), Flow rate: 1 mL\min, Diluent: EtOH

MS (ES^+^) m/z 368 (MH^+^) & LCMS purity 93% (Intermediate **iii**)

MS (ES^+^) m/z 368 (MH^+^) & LCMS purity 89% (Intermediate **iiii**)

Step 4: Preparation of methyl 2-(2-(2-aminoethyl)-6-chloro-1H-benzo[d]imidazol-1-yl) acetate:

To a solution of methyl 2-(2-(2-((tert-butoxycarbonyl)amino)ethyl)-6-chloro-1H-benzo[d]imidazol-1-yl)acetate (500 mg, 1.4 mmol) in dichloromethane (DCM) (5 mL) was added TFA (1mL, 13 mmol) at 0 ºC and then allowed to stir at rt for 2 h. The solvent was removed under reduced pressure, and the residue was dissolved in methanol (20 mL), and basified with amberlyst A-21 ion exchange resin up to pH~8. The resin was filtered and the filtrate was concentrated to obtain methyl 2-(2-(2-aminoethyl)-6-chloro-1H-benzo[d]imidazol-1-yl)acetate (300 mg, 1.104 mmol, 81% yield) as an orange gum. This was carried over to the next step without any further purification. MS (ES^+^) m/z 268 (MH^+^), LCMS purity 98%.

Step 5: Preparation of methyl 2-(2-(2-benzamidoethyl)-6-chloro-1H-benzo[d]imidazol-1-yl) acetate:

To a solution of benzoic acid (164 mg, 1.35 mmol) in dichloromethane (DCM) (10 mL) was added EDC (322 mg, 1.68 mmol), HOBt (257 mg, 1.68 mmol) and TEA (0.234 mL, 1.68 mmol) at rt for 30 min and then added methyl 2-(2-(2-aminoethyl)-6-chloro-1H-benzo[d]imidazol-1-yl)acetate (300 mg, 1.12 mmol). The resulting mixture was stirred at rt for 16 h. The reaction was monitored by TLC (R_f_: 0.45, 5% MeOH in DCM). Upon completion, the reaction was diluted with water (50 mL) and extracted with EtOAc (2 x 50 mL). The combined organics were dried over anhydrous Na_2_SO_4_, filtered and concentrated to obtain methyl 2-(2-(2-benzamidoethyl)-6-chloro-1H-benzo[d]imidazol-1-yl)acetate (300 mg, 0.54 mmol, 48% yield) as colorless crude oil. This crude oil was as such carried over to the next step without any purification. MS (ES^+^) m/z 372 (MH^+^), LCMS purity 66%.

Step 6: Preparation of 2-(2-(2-benzamidoethyl)-6-chloro-1H-benzo[d]imidazol-1-yl) acetic acid:

1N NaOH (5 eq) was added to a solution of methyl 2-(2-(2-benzamidoethyl)-6-chloro-1H-benzo[d]imidazol-1-yl)acetate (300 mg, 0.81 mmol) in tetrahydrofuran (THF) (10 mL) and water (10 mL) at rt and stirred for 3 hr. The reaction was monitored by TLC (R_f_:0.1, 20% MeOH in DCM). Upon completion of the reaction, the reaction mixture was diluted with water (15 mL) and acidified with aq. 5N HCl solution up to pH~3. The aqueous layer was extracted with 10% of MeOH in DCM (5 x 25 mL), and the combined organics were dried over anhydrous Na_2_SO_4_, filtered and concentrated to obtain 2-(2-(2-benzamidoethyl)-6-chloro-1H-benzo[d]imidazol-1-yl)acetic acid (150 mg, 0.330 mmol, 40% yield) as colorless oil. MS (ES^-^) m/z 356 (MH^-^), LCMS purity 78%.

Compound **24**: Preparation of N-(2-(6-chloro-1-(2-((2,3-dihydro-1H-inden-5-yl)amino)-2-oxoethyl)-1H-benzo[d]imidazol-2-yl)ethyl)benzamide:

To a solution of 2-(2-(2-benzamidoethyl)-6-chloro-1H-benzo[d]imidazol-1-yl)acetic acid (150 mg, 0.42 mmol) in N,N-dimethylformamide (DMF) (10 mL) was added EDC (120 mg, 0.63 mmol), HOBt (96 mg, 0.63 mmol) and TEA (0.088 mL, 0.63 mmol) at rt and stirred for 30 min, then 2,3-dihydro-1H-inden-5-amine (67.0 mg, 0.503 mmol) was added. The resulting mixture was stirred at rt for 16 h. The reaction was monitored by TLC (R_f_: 0.3, 5% MeOH in DCM). Upon completion, the reaction was diluted with water (50 mL), extracted with EtOAc (2 x 50 mL), and the combined organics were dried over anhydrous Na_2_SO_4_, filtered and concentrated to obtain the crude, which was triturated with diethyl ether and dried under vacuum to afford N-(2-(6-chloro-1-(2-((2,3-dihydro-1H-inden-5-yl)amino)-2-oxoethyl)-1H-benzo[d]imidazol-2-yl)ethyl)benzamide (95 mg, 0.200 mmol, 47% yield) as an off white solid. ^1^H NMR (DMSO-d_6_, 400 MHz): δ 1.98-2.02 (t, 2H), 2.79-2.81 (t, 4H), 3.16 (t, 2H), 3.75-3.77 (t, 2H), 5.13 (s, 2H), 7.13-7.20 (m, 2H), 7.27-7.29(d, 1H), 7.42-7.53 (m, 4H), 7.59-7.62 (d, 1H), 7.67 (b, 1H), 7.81-7.83 (d, 2H), 8.66 (m, 1H), 10.36 (s, 1H). MS (ES^+^) m/z 473 (MH^+^), LCMS purity 99%.

Synthesis of Compound **25**:

Step-1: Preparation of regiomeric mixture of tert-butyl (3-((2-amino-5-methyl

phenyl)amino)-3-oxopropyl) carbamate and tert-butyl(3-((2-amino-4-methylphenyl)

amino)-3-oxopropyl)carbamate:

To a solution of 4-methylbenzene-1,2-diamine (5.0 g, 41 mmol) in THF (150 mL) was added 3-((tert-butoxycarbonyl)amino)propanoic acid (7.8g, 41 mmol), EDC (11.7g, 61.5 mmol), HOBt (8.3 g, 61. mmol) and TEA (8.5 mL, 61 mmol) at rt. The contents were stirred at rt for 18 h. The reaction mixture was diluted with water (100 mL) and extracted with EtOAc (3 x 100 mL). The combined organics were dried over anhydrous Na_2_SO_4_, filtered and concentrated to obtain crude, which was purified on silica gel (100-200 mesh) column chromatography by using EtOAc as an eluent to afford mixture of regiomers (8g). These isomers were separated by SFC using ethanol as an eluent to afford desired tert-butyl (3-((2-amino-4-methylphenyl)amino)-3-oxopropyl)carbamate (3g) & undesired tert-butyl (3-((2-amino-5-methylphenyl)amino)-3-oxopropyl)carbamate (800 mg) as brown solids. MS (ES^+^) m/z 294 (MH^+^), LCMS purity 99%

Step-2: Preparation of tert-butyl (2-(5-methyl-1H-benzo[d]imidazol-2-yl)ethyl) carbamate:

tert-Butyl (3-((2-amino-4-methylphenyl)amino)-3-oxopropyl)carbamate (500mg, 1.7 mmol) was dissolved in acetic acid (8 mL) and heated at 70 °C for 4 h. After cooling, the solvent was removed under reduced pressure The residue was dissolved in DCM (25 mL) and washed with aqueous sat. NaHCO_3_ solution, separated organic layer was dried over anhydrous Na_2_SO_4_, filtered and concentrated to obtain tert-butyl (2-(5-methyl-1H-benzo[d]imidazol-2-yl)ethyl)carbamate (450 mg, 95 % yield) as pale yellow oil. ^1^H NMR (DMSO-d_6_, 400 MHz): δ 1.42 (s, 9H), 2.45 (s, 3H), 3.09 (t, 2H), 3.63 (t, 2H), 5.20-5.31 (b, 1H), 7.02 (d, 1H), 7.30-7.61 (bs, 2H), 7.58 (b, 1H), 10.2 (b, 1H). MS (ES^+^) m/z 276 (MH^+^), LCMS purity 96%.

Step-3: Preparation of methyl 2-(2-(2-((tert-butoxycarbonyl)amino)ethyl)-5-methyl-1H-benzo[d]imidazol-1-yl)acetate:

To a solution of tert-butyl (2-(5-methyl-1H-benzo[d]imidazol-2-yl)ethyl)carbamate (450 g, 1.6 mmol) in N,N-Dimethylformamide (DMF) (5 mL) was added KOtBu (275mg, 2.45 mmol) followed by methyl bromoacetate (0.18 mL, 2.0 mmol) at 0 ^0^C under nitrogen atmosphere. The resulting mixture was allowed to stir at rt for 2 h. The reaction mixture was diluted with water (50 mL) and extracted with Et_2_O (2 x 30 mL). The combined organics were dried over anhydrous Na_2_SO_4_, filtered and concentrated to methyl 2-(2-(2-((tert-butoxycarbonyl)amino)ethyl)-5-methyl-1H-benzo[d]imidazol-1-yl)acetate (400 mg, 73% yield) as an oil. ^1^H NMR (DMSO-d_6_, 400 MHz): δ 1.42 (s, 9H), 2.52 (s, 3H), 2.95-3.01 (m, 3H), 3.50 (m, 1H), 3.70-3.89 (m, 5H), 4.82 (s, 2H), 5.45-5.49 (b, 1H), 7.05-7.09 (m, 2H), 7.61 (d, 1H). MS (ES^+^) m/z 348 (MH^+^), LCMS purity 73%.

Step-4: Preparation of methyl 2-(2-(2-aminoethyl)-5-methyl-1H-benzo[d]imidazol-1-yl) acetate:

To a solution of methyl 2-(2-(2-((tert-butoxycarbonyl)amino)ethyl)-5-methyl-1H-benzo[d]imidazol-1-yl)acetate in dichloromethane (DCM) (10 mL) was added 4M HCl in 1,4-dioxane (4mL) at 0 ºC for 5 min and then allowed to rt and stirred at for 6 h. The solvent was removed under reduced pressure, and the residue was dissolved in methanol (50 mL), and basified with amberlyst A-21 ion exchange resin up to pH~8. The resin was filtered, and the filtrate was concentrated to obtain methyl 2-(2-(2-aminoethyl)-5-methyl-1H-benzo[d]imidazol-1-yl)acetate (250 mg, 88% yield) as an orange oil, which was carried over to the next step without any further purification. MS (ES^+^) m/z 248 (MH^+^), LCMS purity 75%.

Step-5: Preparation of methyl 2-(2-(2-benzamidoethyl)-5-methyl-1H-benzo[d]imidazol-1-yl)acetate:

To a solution of 2-(2-(2-aminoethyl)-5-methyl-1H-benzo[d]imidazol-1-yl)acetate (250 mg, 1.01 mmol), benzoic acid (136 mg, 1.11 mmol), EDC (290 mg, 1.2 mmol), HOBt (205 mg, 1.16 mmol) in dichloromethane (DCM) (15 mL) was added TEA (0.21 mL, 1.2 mmol) at rt. The resulting mixture was stirred at rt for 18h. Reaction mixture was diluted with water (50 mL) and extracted with EtOAc (2 x 50 mL), combined organics were dried over anhydrous Na_2_SO_4_, filtered and concentrated to obtain crude which was purified by silica gel (100-200 mesh) column chromatography by using 2% of methanol in EtOAc in pet ether as an eluent to afford methyl 2-(2-(2-benzamidoethyl)-5-methyl-1H-benzo[d]imidazol-1-yl)acetate (200mg, 56% yield) as an off white solid. ^1^H NMR (DMSO-d_6_, 400 MHz): δ 2.39 (s, 3H), 3.01-3.03 (t, 2H), 3.65-3.71 (m, 5H), 5.18 (s, 2H), 6.98-7.02 (t, 1H), 7.36-7.58 (m, 5H), 7.82-7.84 (d, 2H), 8.68 (b, 1H). MS (ES^-^) m/z 350 (MH^-^), LCMS purity 77%.

Step-6: Preparation of 2-(2-(2-benzamidoethyl)-5-methyl-1H-benzo[d]imidazol-1-yl) acetic acid:

NaOH (34mg, 0.847 mmol) was added to a solution of methyl 2-(2-(2-benzamidoethyl)-5-methyl-1H-benzo[d]imidazol-1-yl)acetate (200 mg, 0.57 mmol) in tetrahydrofuran (THF) (5 mL) and water (5 mL) at rt and stirred for 4 h. The reaction mixture was diluted with water (15 mL) and extracted with EtOAc (2 x 20 mL) (to remove the impurities). Theaqueous layer was separated and acidified with aq. 5N HCl solution (pH~3), then extracted with 5% of MeOH in DCM (5 x 30 mL)The combined organics were dried over anhydrous Na_2_SO_4_, filtered and concentrated to obtain 2-(2-(2-benzamidoethyl)-5-methyl-1H-benzo[d]imidazol-1-yl)acetic acid (160 mg, 83% yield) as an orange oil. MS (ES^+^) m/z 338 (MH^+^), LCMS purity 89%.

Step-7: Preparation of N-(2-(1-(2-((2,3-dihydro-1H-inden-5-yl)amino)-2-oxoethyl)-5-methyl-1H-benzo[d]imidazol-2-yl)ethyl)benzamide:

To a solution of 2-(2-(2-benzamidoethyl)-5-methyl-1H-benzo[d]imidazol-1-yl)acetic acid (160 mg, 0.4747 mmol) in N,N-dimethylformamide (DMF) (5 mL) was added EDC (136 mg, 0.712 mmol), HOBt (96 mg, 0.71 mmol), TEA (0.1 mL, 0.6 mmol) and 2,3-dihydro-1H-inden-5-amine (69 mg, 0.52 mmol). The resulting mixture was stirred at rt for 16 h. Reaction mixture was diluted with water (30 mL) and extracted with EtOAc (2 x 50 mL) The combined organics were dried over anhydrous Na_2_SO_4_, filtered and concentrated to obtain the crude which was triturated with diethyl ether and dried under vacuum to afford N-(2-(1-(2-((2,3-dihydro-1H-inden-5-yl)amino)-2-oxoethyl)-5-methyl-1H-benzo[d]imidazol-2-yl)ethyl)benzamide (110 mg, 51% yield) as an off white solid. ^1^H NMR (DMSO-d_6_, 400 MHz): δ 1.98-2.02 (t, 2H), 2.52 (s, 3H), 2.79-2.81 (t, 4H), 3.16 (t, 2H), 3.77-3.80 (t, 2H), 5.13 (s, 2H), 6.97-7.02 (t, 1H), 7.13-7.17 (d, 1H), 7.22-7.39(m, 2H), 7.42-7.58 (m, 5H), 7.81-7.83 (d, 2H), 8.66 (m, 1H), 10.36 (s, 1H). MS (ES^+^) m/z 453 (MH^+^), HPLC purity 97%.

Synthesis of Compound **26**:

Step-1: Preparation of methyl 2-(2-(2-aminoethyl)-5-methoxy-1H-benzo[d]imidazol-1-yl) acetate:

To a solution of methyl 2-(2-(2-((tert-butoxycarbonyl)amino)ethyl)-5-methoxy-1H-benzo[d]imidazol-1-yl)acetate (150 mg, 0.413 mmol) in dichloromethane (DCM) (5mL) was added TFA (1 mL, 13 mmol) at 0 ºC and then allowed to rt for 2 h. The solvent was removed under reduced pressure, the residue was dissolved in methanol (20 mL), and basified with amberlyst A-21 ion exchange resin up to pH~8. Theresin was filtered and thefiltrate was concentrated to obtain methyl 2-(2-(2-aminoethyl)-5-methoxy-1H-benzo[d]imidazol-1-yl)acetate (100 mg, 0.361 mmol, 87% yield) as an orange gum, which was carried over to the next step without any further purification. MS (ES^+^) m/z 264 (MH^+^), LCMS purity 95%.

Step-2:Preparation of methyl 2-(2-(2-benzamidoethyl)-5-methoxy-1H-benzo[d] imidazol-1-yl)acetate:

To a solution of benzoic acid (56 mg, 0.46 mmol) in dichloromethane (DCM) (10 mL) was added EDC (109 mg, 0.570 mmol), HOBt (87 mg, 0.57 mmol), TEA (0.079 mL, 0.57 mmol) at rt and stirred for 30 min and then methyl 2-(2-(2-aminoethyl)-5-methoxy-1H-benzo[d]imidazol-1-yl)acetate (100 mg, 0.380 mmol) was added. The resulting mixture was stirred at rt for 16 h. The reaction was monitored by TLC (R_f_: 0.45, 5% MeOH in DCM). Upon completion, the reaction was diluted with water (50 mL) and extracted with EtOAc (2 x 50 mL). The combined organics were dried over anhydrous Na_2_SO_4_, filtered and concentrated to obtain methyl 2-(2-(2-benzamidoethyl)-5-methoxy-1H-benzo[d]imidazol-1-yl)acetate (100 mg, 0.247 mmol, 65% yield) as colorless oil, which was carried over to the next step without any further purification. MS (ES^+^) m/z 368 (MH^+^), LCMS purity 90%.

Step-3: Preparation of 2-(2-(2-benzamidoethyl)-5-methoxy-1H-benzo[d]imidazol-1-yl) acetic acid:

1N NaOH (5 eq) was added to a solution of methyl 2-(2-(2-benzamidoethyl)-5-methoxy-1H-benzo[d]imidazol-1-yl)acetate (100 mg, 0.27 mmol) in tetrahydrofuran (THF) (5 mL) and water (5 mL) at rt for 3 h. The reaction was monitored by TLC (R_f_:0.1, 20% MeOH in DCM). Upon completion, the reaction was diluted with water (10 mL) and extracted with EtOAc (to remove the impurities). The separated aqueous layer was acidified with aq. 5N HCl solution (pH~2) and extracted with 10% MeOH in DCM (5 x 25 mL). Thehe combined organics were dried over anhydrous Na_2_SO_4_, filtered and concentrated to obtain 2-(2-(2-benzamidoethyl)-5-methoxy-1H-benzo[d]imidazol-1-yl)acetic acid (60 mg, 0.166 mmol, 61% yield) as an colorless oil. MS (ES^+^) m/z 354 (MH^+^), LCMS purity 97%.

Step-4: Preparation of N-(2-(1-(2-((2,3-dihydro-1H-inden-5-yl)amino)-2-oxoethyl)-5-methoxy-1H-benzo[d]imidazol-2-yl)ethyl)benzamide:

To a solution of 2-(2-(2-benzamidoethyl)-5-methoxy-1H-benzo[d]imidazol-1-yl)acetic acid (60 mg, 0.170 mmol) in N,N-dimethylformamide (DMF) (5 mL) was added EDC (49 mg, 0.26 mmol), HOBT (39 mg, 0.26 mmol) and TEA (0.035 mL, 0.26 mmol) at rt and stirred for 30 min and then 2,3-dihydro-1H-inden-5-amine (27 mg, 0.20 mmol) was added. The resulting mixture was stirred at rt for 10 h. The reaction was monitored by TLC (R_f_: 0.3, 5% MeOH in DCM). Upon completion, the reaction was diluted with water (50 mL) and extracted with EtOAc (2 x 50 mL). The combined organics were dried over anhydrous Na_2_SO_4_, filtered and concentrated to obtain the crude, which was triturated with diethyl ether and dried under vacuum to afford N-(2-(1-(2-((2,3-dihydro-1H-inden-5-yl)amino)-2-oxoethyl)-5-methoxy-1H-benzo[d]imidazol-2-yl)ethyl)benzamide (15 mg, 0.031 mmol, 18% yield) as an off white solid. ^1^H NMR (DMSO-d_6_, 400 MHz): δ 1.98-2.02 (t, 2H), 2.75-2.85 (m, 4H), 3.18-3.22 (t, 2H), 3.75-3.79 (m, 5H), 5.13 (s, 2H), 6.81-6.83 (d, 1H), 7.12-7.16 (m, 1H), 7.25-7.35 (m, 2H), 7.42-7.53 (m, 4H), 7.81-7.84 (m, 2H), 7.82-7.85 (d, 2H), 8.62-8.68 (m, 1H), 10.35 (s, 1H). MS (ES^+^) m/z 469 (MH^+^), LCMS purity 97%.

Synthesis of Compound **27**:

Step-1: Preparation of regiomeric mixture of tert-butyl (3-((2-amino-4-methoxyphenyl)amino)-3-oxopropyl) carbamate & tert-butyl (3-((2-amino-5-methoxy

phenyl)amino)-3-oxopropyl)carbamate:

To a solution of 3-((tert-butoxycarbonyl)amino)propanoic acid (1.37 g, 7.24 mmol) in N,N-Dimethylformamide (DMF) (25 mL) was added TEA (3.03 mL, 21.7 mmol), EDC (2.08 g, 10.9 mmol), HOBT (1.66 g, 10.9 mmol) at rt and stirred for 1h and then 4-methoxybenzene- 1,2-diamine (1.0 g, 7.2 mmol) was added. The resulting mixture was stirred at rt for 18 h. Reaction mixture was diluted with water (100 mL) and extracted with EtOAc (2 x 50 mL). The combined organics were dried over anhydrous Na_2_SO_4_, filtered and concentrated to obtain as an orange solid (crude), which was purified by silica gel (100-200 mesh) column chromatography by using 60% EtOAc in pet ether as an eluent to afford tert-butyl (3-((2-amino-4-methoxyphenyl)amino)-3-oxopropyl)carbamate compound with tert-butyl (3-((2-amino-5-methoxyphenyl)amino)-3-oxopropyl)carbamate (1:1) (350 mg, 0.57 mmol, 7% yield) as an off white solid.

Step-2: Preparation of (1: 1) tautomeric mixture of tert-butyl (2-(5-methoxy-1H-benzo[d]imidazol-2-yl)ethyl) carbamate & tert-butyl (2-(6-methoxy-1H-benzo[d]

imidazol-2-yl)ethyl)carbamate:

tert-Butyl (3-((2-amino-4-methoxyphenyl)amino)-3-oxopropyl)carbamate with tert-butyl (3-((2-amino-5-methoxyphenyl)amino)-3-oxopropyl)carbamate (1:1) (350 mg, 0.57 mmol) was dissolved in acetic acid (10 mL) and heated at 70 °C for 3hr. After cooling, the solvent was removed under reduced pressure. The residue was dissolved in DCM (25 mL) and washed with sat. NaHCO_3_, dried over anhydrous Na_2_SO_4_, filtered and concentrated to obtain mixture(1: 1) of crude tert-butyl (2-(5-methoxy-1H-benzo[d]imidazol-2-yl)ethyl)carbamate and tert-butyl (2-(6-methoxy-1H-benzo[d]imidazol-2-yl)ethyl)carbamate (1:1) (300 mg, 0.52 mmol, 91% yield) as an orange gum, which was as such carried over to the next step, without any purification and analysis.

Step-3: Preparation of regiomeric mixture of methyl 2-(2-(2-((tert-butoxy

carbonyl)amino)ethyl)-6-methoxy-1H-benzo[d]imidazol-1-yl)acetate & methyl 2-(2-(2-((tert-butoxycarbonyl)amino)ethyl)-5-methoxy-1H-benzo[d]imidazol-1-yl)acetate:

To a solution of tert-butyl (2-(5-methoxy-1H-benzo[d]imidazol-2-yl)ethyl)carbamate with tert-butyl (2-(6-methoxy-1H-benzo[d]imidazol-2-yl)ethyl)carbamate (1:1) (300 mg, 0.52 mmol) in N,N-dimethylformamide (DMF) (5 mL) was added KOtBu (87 mg, 0.77 mmol) and methyl bromoacetate (0.057 mL, 0.62 mmol) at 0 ºC under nitrogen atmosphere. The resulting mixture was stirred at rt for 3 h. The reaction mixture was diluted with water (30 ml) and extracted with EtOAc (2 x 30 mL). The combined organics were dried over anhydrous Na_2_SO_4_, filtered and concentrated to obtain crude, which was purified by silica gel (100-200 mesh) column chromatography by using 60% EtOAc in pet ether as an eluent to afford the desired mixture as an off white solid (180 mg, regiomers). which was purified on preparative HPLC by using Hexane: ethanol (7:3) as an eluent to afford methyl 2-(2-(2-((tert-butoxycarbonyl)amino)ethyl)-6-methoxy-1H-benzo[d]imidazol-1-yl)acetate (150 mg, 0.36 mmol, 69% yield) and methyl 2-(2-(2-((tert-butoxycarbonyl)amino)ethyl)-5-methoxy-1H-benzo[d]imidazol-1-yl)acetate (150 mg, 0.39 mmol, 76% yield) as orange gum.

Following conditions were used to separate the regiomers.

Column: Chiral pak IC (4.6 x 250mm) 5μ

Mobile phase: A: Hexane, B: EtOH

ISO: A: B (70:30)

Flow rate: 1 mL\min

Diluent: EtOH

Compound A - MS (ES^+^) m/z 363.88(MH^+^) & LCMS purity 87%

Compound B - MS (ES^+^) m/z 363.9(MH^+^) & LCMS purity 94%

Step-4: Preparation of methyl 2-(2-(2-aminoethyl)-6-methoxy-1H-benzo[d]imidazol-1-yl)acetate:

To a solution of methyl 2-(2-(2-((tert-butoxycarbonyl)amino)ethyl)-6-methoxy-1H-benzo[d]imidazol-1-yl)acetate (150 mg, 0.41 mmol) in dichloromethane (DCM) (5 mL) was added TFA (1.0 mL, 13 mmol) at 0 ºC and then allowed to rt for 2 h. . The solvent was removed under reduced pressure, and the residue was dissolved in methanol (20 mL) and basified with amberlyst A-21 ion exchange resin up to pH~8. The resin was filtered and filtrate was concentrated to obtain methyl 2-(2-(2-aminoethyl)-6-methoxy-1H-benzo[d]imidazol-1-yl)acetate (100 mg, 0.345 mmol, 84% yield) as an orange gum, which was carried over to the next step without any further purification. MS (ES^+^) m/z 264 (MH^+^), LCMS purity 90%.

Step-5: Preparation of methyl 2-(2-(2-benzamidoethyl)-6-methoxy-1H-benzo[d] imidazol-1-yl)acetate:

To a solution of benzoic acid (55.7 mg, 0.456 mmol) in dichloromethane (DCM) (10 mL) was added EDC (109 mg, 0.57 mmol), HOBt (87 mg, 0.57 mmol) and TEA (0.079 mL, 0.57 mmol) at rt for 30 min and then methyl 2-(2-(2-aminoethyl)-6-methoxy-1H-benzo[d]imidazol-1-yl)acetate (100 mg, 0.380 mmol) was added. The resulting mixture was stirred at rt for 16 h. The reaction was monitored by TLC (R_f_: 0.45, 5% MeOH in DCM). Upon completion, the reaction was diluted with water (50 mL), extracted with EtOAc (2 x 50 mL). The combined organics were dried over anhydrous Na_2_SO_4_, filtered and concentrated to obtain methyl 2-(2-(2-benzamidoethyl)-6-methoxy-1H-benzo[d]imidazol-1-yl)acetate (100 mg, 0.26 mmol, 69% yield) as colorless oil, which was carried over to the next step without any further purification. ^1^H NMR (DMSO-d_6_, 400 MHz): δ 3.07-3.11 (t, 2H), 3.72 (s, 3H), 3.82 (s, 3H), 4.07 (t, 2H), 4.82 (s, 2H), 6.72 (d, 1H), 6.94 (m, 1H), 7.41-7.52 (m, 3H), 7.63 (d, 2H), 7.78 (d, 2H). MS (ES^+^) m/z 368 (MH^+^), LCMS purity 96%.

Step-6: Preparation of 2-(2-(2-benzamidoethyl)-6-methoxy-1H-benzo[d]imidazol-1-yl) acetic acid :

1N NaOH (5 eq) was added to a solution of methyl 2-(2-(2-benzamidoethyl)-6-methoxy-1H-benzo[d]imidazol-1-yl)acetate (100 mg, 0.272 mmol) in tetrahydrofuran (THF) (5 mL) and water (5.0 mL) at rt and stirred for 3 hr. The reaction was monitored by TLC (R_f_:0.1, 20% MeOH in DCM). Upon completion, the reaction was diluted with water (10 mL) and extracted with ethyl acetate (to remove the impurities). The separated aqueous layer was acidified with aq. 5N HCl solution (pH~3), then extracted with 10% MeOH in DCM (5 x 25 mL). The combined organics were dried over anhydrous Na_2_SO_4_, filtered and concentrated to obtain 2-(2-(2-benzamidoethyl)-6-methoxy-1H-benzo[d]imidazol-1-yl)acetic acid (60 mg, 0.16 mmol, 60% yield) as an colorless oil. MS (ES^+^) m/z 354 (MH^+^), LCMS purity 96%.

Step-7: Preparation of N-(2-(1-(2-((2,3-dihydro-1H-inden-5-yl)amino)-2-oxoethyl)-6-methoxy-1H-benzo[d]imidazol-2-yl)ethyl)benzamide:

To a solution of 2-(2-(2-benzamidoethyl)-6-methoxy-1H-benzo[d]imidazol-1-yl)acetic acid (60 mg, 0.170 mmol) in N,N-dimethylformamide (DMF) (5 mL) was added EDC (49 mg, 0.26 mmol), HOBT (39 mg, 0.26 mmol) and TEA (0.035 mL, 0.26 mmol) at rt for 30 min and then 2,3-dihydro-1H-inden-5-amine (27 mg, 0.20 mmol) was added. The resulting mixture was stirred at rt for 10 h. the reaction was monitored by TLC (R_f_: 0.3, 5% MeOH in DCM). Upon completion, the reaction was diluted with water (50 mL), extracted with EtOAc (2 x 50 mL) The combined organics were dried over anhydrous Na_2_SO_4_, filtered and concentrated to obtain the crude, which was triturated with diethyl ether and dried under vacuum to afford N-(2-(1-(2-((2,3-dihydro-1H-inden-5-yl)amino)-2-oxoethyl)-6-methoxy-1H-benzo[d]imidazol-2-yl)ethyl)benzamide (20 mg, 0.042 mmol, 24% yield) as an off white solid. ^1^H NMR (DMSO-d_6_, 400 MHz): δ 1.98-2.02 (t, 2H), 2.79-2.81 (m, 4H), 3.07-3.09 (t, 2H), 3.75-3.77 (t, 5H), 5.07 (s, 2H), 6.79-6.81 (m, 1H), 7.07-7.08 (m, 1H), 7.13-7.15 (m, 1H), 7.27-7.29 (m, 1H), 7.42-7.52 (m, 5H), 7.81-7.83 (d, 2H), 8.66-8.67 (m, 1H), 10.36 (s, 1H). MS (ES^+^) m/z 469 (MH^+^), LCMS purity 99%.

Synthesis of Compound **28**:

Step-1: Preparation of tert-butyl (3-((2-amino-3-methylphenyl)amino)-3-oxopropyl) carbamate:

To a solution of 3-methylbenzene-1,2-diamine (7.2 g, 59 mmol) in THF (220 mL) was added 3-((tert-butoxycarbonyl)amino)propanoic acid (11.1 g, 59.0 mmol), EDC (17 g, 88 mmol), HOBt (12.0 g, 88.5 mmol) and TEA (12.0 mL, 88.5 mmol) at rt. The resulting mixture was stirred at rt for 18 h. The reaction mixture was diluted with water (150 mL) and extracted with EtOAc (2 x 150 mL). Thecombined organics were dried over anhydrous Na_2_SO_4_, filtered and concentrated to obtain crude, which was purified by silica gel (100-200 mesh) column chromatography using 30% EtOAc in pet ether as an eluent to afford tert-butyl (3-((2-amino-3-methylphenyl)amino)-3-oxopropyl)carbamate (13 g, 75% yield) as an off white solid. ^1^H NMR (DMSO-d_6_, 400 MHz): δ 1.42 (s, 9H), 2.08 (s, 3H), 2.45 (t, 2H), 3.25 (t, 2H), 4.58 (s, 2H), 6.48-6.52 (t, 1H), 6.81-6.85 (d, 2H), 7.05 (d, 1H), 9.05 (s, 1H). MS (ES^+^) m/z 294 (MH^+^), LCMS purity 82%.

Step-2: Preparation of tert-butyl (2-(4-methyl-1H-benzo[d]imidazol-2-yl)ethyl) carbamate:

tert-Butyl (3-((2-amino-3-methylphenyl)amino)-3-oxopropyl)carbamate (3.0 g, 10.2 mmol) was dissolved in acetic acid (30 mL) and heated at 70 °C for 4 h, then cooled down to rt. The solvent was removed under reduced pressure. The residue was dissolved in DCM (25 mL) and washed with sat. NaHCO_3_ solution, separated organic layer was dried over anhydrous Na_2_SO_4_, filtered and concentrated to obtain tert-butyl (2-(4-methyl-1H-benzo[d]imidazol-2-yl)ethyl)carbamate (2.7 g, 96% yield) as an orange oil. ^1^HNMR (DMSO-d_6_, 400 MHz): δ 1.42 (s, 9H), 2.45 (s, 3H), 3.19 (t, 2H), 3.70 (t, 2H), 5.15-5.21 (b, 1H), 7.02 (t, 1H), 7.11-7.19 (b, 2H), 7.58 (b, 1H), 10.2 (b, 1H). MS (ES^+^) m/z 276 (MH^+^), LCMS purity 84%.

Step-3: Preparation of methyl 2-(2-(2-((tert-butoxycarbonyl)amino)ethyl)-4-methyl-1H-benzo[d]imidazol-1-yl)acetate:

To a solution of tert-butyl (2-(4-methyl-1H-benzo[d]imidazol-2-yl)ethyl)carbamate (2.7 g, 9.8 mmol) in N,N-dimethylformamide (DMF) (25 mL) was added KOtBu (1.77g, 14.7 mmol) and methyl bromoacetate (0.99 mL, 10.8 mmol) at 0 ºC under nitrogen atmosphere. The resulting mixture was allowed to rt and stirred for 4 hr. Reaction mixture was diluted with water (50 mL) and extracted with Et_2_O (2 x 50 mL). The combined organics were dried over anhydrous Na_2_SO_4_, filtered and concentrated to obtain 2-(2-(2-((tert-butoxycarbonyl)amino)ethyl)-4-methyl-1H-benzo[d]imidazol-1-yl)acetate (2.5g, 73% yield) as an oil. ^1^H NMR (DMSO-d_6_, 400 MHz): δ 1.42 (s, 9H), 2.63 (s, 3H), 3.05 (t, 2H), 3.70 (t, 2H), 3.79 (s, 3H), 4.82 (s, 1H), 5.45-5.49 (b, 1H), 7.01-7.05 (m, 2H), 7.18 (m, 2H). MS (ES^+^) m/z 348 (MH^+^), LCMS purity 87%.

Step-4: Preparation of methyl 2-(2-(2-aminoethyl)-4-methyl-1H-benzo[d]imidazol-1-yl)

acetate:

To a solution of methyl 2-(2-(2-((tert-butoxycarbonyl)amino)ethyl)-4-methyl-1H-benzo[d] imidazol-1-yl)acetate (2.5g, 7.2 mmol) in Dichloromethane (120 mL) was added 4M HCl in 1,4-dioxane (20mL) at 0º C for 5min and allowed to rt for 4h. The solvent was evaporated under reduced pressure, the residue was dissolved in methanol (50 mL), basified with amberlyst A-21 ion exchange resin up to pH~8, filtered the resin and filtrate was concentrated to obtain methyl 2-(2-(2-aminoethyl)-4-methyl-1H-benzo[d]imidazol-1-yl)acetate (1.5g, 84% yield) as an orange oil, which was carried over to next step without any purification. MS (ES^+^) m/z 248 (MH^+^), LCMS purity 88%.

Step-5: Preparation of methyl 2-(2-(2-benzamidoethyl)-4-methyl-1H-benzo[d]imidazol-1-yl)acetate:

To a solution of methyl 2-(2-(2-aminoethyl)-4-methyl-1H-benzo[d]imidazol-1-yl)acetate (1.5g, 6.0 mmol), benzoic acid (815 mg, 6.68 mmol), EDC (1.73g, 9.10 mmol), HOBt (1.30g, 9.10 mmol) in N,N-Dimethylformamide (25 mL) was added TEA (1.26 mL, 9.10 mmol) at rt. The resulting mixture was stirred at rt for 16h. The reaction mixture was diluted with water (200 mL) and extracted with EtOAc (2 x 100 mL). The combined organics were dried over anhydrous Na_2_SO_4_, filtered and concentrated to obtain crude, which was triturated with diethyl ether to afford methyl 2-(2-(2-benzamidoethyl)-4-methyl-1H-benzo[d]imidazol-1-yl)acetate (1g, 46% yield) as brown solid. This was carried over to the next step without any further purification. MS (ES^+^) m/z 352 (MH^+^), LCMS purity 70%.

Step-6: Preparation of 2-(2-(2-benzamidoethyl)-4-methyl-1H-benzo[d]imidazol-1-yl) acetic acid:

To a solution of methyl 2-(2-(2-benzamidoethyl)-4-methyl-1H-benzo[d]imidazol-1-yl)acetate (1.0g, 2.8 mmol) in Tetrahydrofuran (THF) (20 mL) and water (20 mL) was added NaOH (171mg, 4.27 mmol) at 0°C. The contents were stirred at rt for 4h. The reaction mixture was diluted with water (50 mL) and extracted with EtOAc (2 x 50 mL) (to remove the impurities) The separated aqueous layer was acidified with aq. 5N HCl solution (PH~3), then extracted with 5% of MeOH in DCM (3 x 50 mL) The combined organics were dried over anhydrous Na2SO4, filtered and concentrated to obtain 2-(2-(2-benzamidoethyl)-4-methyl-1H-benzo[d]imidazol-1-yl)acetic acid (700 mg, 72% yield) as an orange oil. ^1^H NMR (DMSO-d_6_, 400 MHz): δ 2.63 (s, 3H), 3.42 (t, 2H), 3.79 (t, 2H), 5.42 (s, 2H), 7.32-7.56 (m, 5H), 7.68-7.71 (d, 1H), 7.85-7.89 (d, 1H), 8.91 (b, 1H). MS (ES^+^) m/z 338 (MH^+^), LCMS purity 81%.

Step-7: Preparation of N-(2-(1-(2-((2,3-dihydro-1H-inden-5-yl)amino)-2-oxoethyl)-4-methyl-1H-benzo[d]imidazol-2-yl)ethyl)benzamide:

To a solution of 2-(2-(2-benzamidoethyl)-4-methyl-1H-benzo[d]imidazol-1-yl)acetic acid (700 mg, 2.07 mmol) in N,N-Dimethylformamide (DMF) (15 mL) was added EDC (595 mg, 3.11 mmol), HOBt (420 mg, 3.11 mmol), TEA (0.43 mL, 3.11 mmol) and 2,3-dihydro-1H-inden-5-amine (303 mg, 2.284 mmol). The contents were stirred at rt for 16h. The reaction mixture was diluted with water (50 mL) and extracted with EtOAc (3 x 50 mL). The combined organics were dried over anhydrous Na2SO4, filtered and concentrated to obtain the crude product, which was triturated with diethyl ether and dried under vacuum to afford N-(2-(1-(2-((2,3-dihydro-1H-inden-5-yl)amino)-2-oxoethyl)-4-methyl-1H-benzo[d]imidazol-2-yl)ethyl)benzamide (300 mg, 32% yield) as an off white solid. ^1^H NMR (DMSO-d_6_, 400 MHz): δ 1.98-2.02 (t, 2H), 2.52 (s, 3H), 2.79-2.81 (t, 4H), 3.16 (t, 2H), 3.77-3.80 (t, 2H), 5.13 (s, 2H), 6.97-7.06 (d, 1H), 7.08-7.12(t, 1H), 7.12-7.15 (d, 1H), 7.25-7.28 (t, 2H), 7.43-7.56 (m, 4H), 7.82-7.86 (d, 2H), 8.66 (m, 1H), 10.36 (s, 1H). MS (ES^+^) m/z 453 (MH^+^), LCMS purity 97%.

Synthesis of Compound **29**:

Step-1: Preparation of 2-(2-(2-(N-methylbenzamido)ethyl)-1H-benzo[d]imidazol-1-yl)acetic acid:

To a stirred solution of methyl 2-(2-(2-benzamidoethyl)-1H-benzo[d]imidazol-1-yl)acetate (500 mg, 1.48 mmol) in Tetrahydrofuran (THF) (40 mL) at 0°C was added sodium hydride (89 mg, 3.71 mmol) and was stirred at rt for 30 min. Methyl iodide (0.185 mL, 2.96 mmol) was added drop wise at rt and the reaction mixture was stirred for 16hr. TLC indicated completion of the reaction. The reaction mixture was quenched with water (10 mL) and concentrated to afford 2-(2-(2-(N-methylbenzamido)ethyl)-1H-benzo[d]imidazol-1-yl)acetic acid (500 mg, 0.148 mmol, 10% yield). MS (ES^+^) m/z 338 (MH^+^), LCMS purity: 97%.

Step-2: Preparation of N-(2-(1-(2-((2,3-dihydro-1H-inden-5-yl)amino)-2-oxoethyl)-1H-benzo[d]imidazol-2-yl)ethyl)-N-methylbenzamide:

To a stirred solution of 2-(2-(2-(N-methylbenzamido)ethyl)-1H-benzo[d]imidazol-1-yl)acetic acid (500 mg, 1.48 mmol) in N,N-Dimethylformamide (DMF) (10 mL) was added 2,3-dihydro-1H-inden-5-amine (296 mg, 2.22 mmol), EDC (426 mg, 2.23 mmol), HOBT (340 mg, 2.22 mmol) and TEA (0.620 mL, 4.45 mmol) . The resulting reaction mixture was stirred at rt for 16 h until completion of the reaction. The reaction mixture was diluted with EtOAc (300 mL) and Water (100 mL) extracted and separated the layers, organic layer was dried Na_2_SO_4_, filtered and concentrated to afford the crude material. The crude product was added to a silica gel (230-400 mesh) column and eluted with 2% MeOH in DCM. The collected product fractions were concentrated to give N-(2-(1-(2-((2,3-dihydro-1H-inden-5-yl)amino)-2-oxoethyl)-1H-benzo[d]imidazol-2-yl)ethyl)-N-methylbenzamide (80 mg, 0.172 mmol, 11% yield). ^1^H NMR (DMSO-d_6_, 400 MHz) δ: 1.8-2.0 (m, 2H), 2.8-3.0 (m, 4H), 3.0-3.2 (m, 5H), 3.6-3.8 (s, 1H), 3.8-4.0 (s, 1H), 4.8-5.0 (s, 1H), 5.0-5.2 (s, 1H), 7.2-7.7 (m, 12H), 10.3-10.4 (d, 1H). MS (ES^+^) m/z 453 (MH^+^), LCMS purity: 97%.

Synthesis of Compound **30**:

Step-1: Preparation of N-(2-(1-(2-((2,3-dihydro-1H-inden-5-yl)amino)-2-oxoethyl)-1H-benzo[d]imidazol-2-yl)ethyl)benzamide:

To a solution of 2-(2-(2-benzamidoethyl)-1H-benzo[d]imidazol-1-yl) acetic acid (500 mg, 1.54 mmol) in DMF (15 mL) was added 2,3-dihydro-1H-inden-5-amine (164 mg, 1.23 mmol), EDC. HCl, (445 mg, 2.31 mmol), HOBt (313 mg, 2.31 mmol) and TEA (0.387 mL, 2.78 mmol) at rt. The contents were stirred at rt for 14 h and the reaction was monitored by TLC until completion. The reaction mixture was poured on to ice water, a precipitate was formed and was filtered, washed with cold water, ether and dried under high vacuum to obtain N-(2-(1-(2-((2,3-dihydro-1H-inden-5-yl)amino)-2-oxoethyl)-1H-benzo[d]imidazol-2-yl)ethyl)benzamide (350 mg, 0.799 mmol, 51% yield) as an off white solid. ^1^H NMR (DMSO-d6 400 MHz) δ: 1.92-2.02 (m, 3H), 2.72-2.83 (m, 4H), 3.08-3.18 (t, 2H), 3.72-3.82 (q, 2H), 5.16 (s, 2H), 7.12-7.22 (m, 3H), 7.23-7.3(d, 1H), 7.4-7.58 (m, 5H), 7.58-7.62 (d, 1H), 7.8-7.85 (d, 2H), 8.66 (b, 1H), 10.4 (s, 1H). MS (ES^+^) m/z 439 (MH^+^), LCMS purity: 95%.

Step-2: Preparation of N-(2-(1-(2-((2,3-dihydro-1H-inden-5-yl)(methyl)amino)-2-oxo

ethyl)-1H-benzo[d]imidazol-2-yl)ethyl)-N-methylbenzamide:

To a solution of N-(2-(1-(2-((2,3-dihydro-1H-inden-5-yl)amino)-2-oxoethyl)-1H-benzo[d]

imidazol-2-yl)ethyl)benzamide (150 mg, 0.344 mmol) in THF (5 mL) & DMF (2 mL) at 0°C was added NaH (41.3 mg, 1.03 mmol) in small portions and stirred at 0°C for 30 min and then at rt for 30min. Then, methyl iodide (0.0429 mL, 0.689 mmol) was added drop wise at 0°C and stirred for 1h then allowed to rt for 3h. The reaction was monitored by TLC. On completion, the reaction mixture was quenched with ice water (15 mL) and extracted with EtOAc (3 x 20 mL). The combined organics were washed with brine, dried over sodium sulfate, filtered and concentrated under reduced pressure to afford N-(2-(1-(2-((2,3-dihydro-1H-inden-5-yl)(methyl)amino)-2-oxoethyl)-1H-benzo[d]imidazol-2-yl)ethyl)-N-methylbenzamide (55 mg, 0.11 mmol, 34% yield) as an off white powder. ^1^H NMR (CDCl_3_-d6 400 MHz) δ: 2.1-2.2 (m, 2H), 2.82-3.0 (m, 7H), 3.1-3.3 (m, 5H), 3.8-4 (m, 2H), 4.8 (s, 1H), 6.7 (s, 1H), 6.95-7.12 (m, 2H), 7.14-7.3 (m, 4H), 7.3-7.4 (m, 5H), 7.6-7.7 (b, 1H). MS (ES^+^) m/z 467 (MH^+^), LCMS purity: 97%, HPLC purity: 95%.

Synthesis of Compound **31**:

Step-1: Preparation of N-methyl-2,3-dihydro-1H-inden-5-amine:

To a solution of 2,3-dihydro-1H-inden-5-amine (500 mg, 3.75 mmol) in 1,4-dioxane (10 mL) was added K_2_CO_3_ (518 mg, 3.76 mmol) stirred for 30 minutes at rt. To this mixture was added iodomethane (266 mg, 1.88 mmol) and the reaction was stirred for 4 hr at rt. The solvent was removed to afford the crude material which was diluted with water (30 mL) and ethyl acetate (50 mL) and the layers were separated. The organic layer was dried over Na_2_SO_4_, filtered and concentrated under vacuum. The crude compound was purified by silica gel (230-400 mesh) column chromatography. The compound was eluted with 5% EtOAc in hexane to get 120 mg of N-methyl-2,3-dihydro-1H-inden-5-amine(yield: 21%) as a colourless liquid. MS (ES^+^) m/z 148 (MH^+^), LCMS purity: 89%.

Step-2: Preparation of N-(2-(1-(2-((2,3-dihydro-1H-inden-5-yl)(methyl)amino)-2-oxo

ethyl)-1H-benzo[d]imidazol-2-yl)ethyl)benzamide:

To a solution of N-methyl-2,3-dihydro-1H-inden-5-amine (100 mg, 0.680 mmol) in N,N-Dimethylformamide (DMF) (5 mL) was added 2-(2-(2-benzamidoethyl)-1H-benzo[d]imidazol-1-yl)acetic acid (219 mg, 0.680 mmol), EDC (194 mg, 1.01 mmol), HOBT (137 mg, 1.01 mmol) and TEA (206 mg, 2.03 mmol). The reaction mixture was stirred at rt for 16 h and monitored by TLC. After completion of the reaction, the mixture was diluted with water (20 mL) and EtOAc (50 mL) and the layers were separated. The organic layer was washed with a saturated NaHCO_3_ solution (20 mL) and dried over anhydrous Na_2_SO_4_, filtered and concentrated to get the crude material. The crude compound was purified by silica gel (100-200 mesh) column chromatography and was eluted with 3% MeOH in DCM to give 80 mg of N-(2-(1-(2-((2,3-dihydro-1H-inden-5-yl)(methyl)amino)-2-oxoethyl)-1H-benzo[d]imidazol-2-yl)ethyl)benzamide (yield: 26%) as a yellow solid. ^1^H NMR (DMSO-d_6_, 400 MHz) δ: 2.0-2.1 (t, 2H), 2.85-2.90 (m, 4H), 2.90-3.0 (t, 2H), 3.1-3.15 (s, 3H), 3.6-3.70 (m, 2H), 4.7-4.80 (s, 2H), 7.1-7.2 (m, 2H), 7.2-7.3 (m, 1H), 7.3-7.4 (m, 2H), 7.4- 7.5 (m, 3 H), 7.5- 7.6 (m, 2 H), 7.8-7.85 (d, 2H), 8.6-8.7 (broad, 1H). MS (ES^+^) m/z 453 (MH^+^), LCMS purity: 96%.

Synthesis of Compounds **32-37**:

Step-1: Preparation of methyl 2-(2-(2-benzamidoethyl)-1H-benzo[d]imidazol-1-yl)

acetate:

To a solution of methyl 2-(2-(2-aminoethyl)-1H-benzo[d]imidazol-1-yl)acetate (1.40g, 6.00 mmol) in N,N-Dimethylformamide (DMF) (30 mL) was added EDC (1.72 g, 9.00 mmol), HOBt (1.37 g, 9.00 mmol) and TEA (1.6 mL, 12 mmol) & benzoic acid (0.88 g, 7.20 mmol) at rt and stirred for 16 hr. The reaction was monitored by TLC(R_f_: 0.3, 5% MeOH in DCM), and on completion of the reaction, water (100 mL) was added to the mixture. The aqueous layer was extracted with EtOAc (2 x 50 mL). The separated organic layers were then combined, dried over anhydrous Na_2_SO_4_, filtered and concentrated to obtain the crude material. The crude material was triturated with diethyl ether and dried under vacuum to afford methyl 2-(2-(2-benzamidoethyl)-1H-benzo[d]imidazol-1-yl)acetate (1.4g, 4.12 mmol, 68% yield) as a white solid. ^1^H NMR (DMSO-d_6_, 400 MHz): δ 3.01-3..09 (t, 2H), 3.65-3.75 (m, 5H), 5.21 (s, 2H), 7.15-7.20(m, 2H), 7.42-7.56 (m, 5H), 7.58-7.62 (m, 1H), 7.82-7.88 (d, 2H), 8.66-8.72 (t, 1H). MS (ES^+^) m/z 338 (MH^+^), LCMS purity 99%.

Step-2 & 3: Preparation of 2-(2-(2-(N-methylbenzamido)ethyl)-1H-benzo[d]imidazol-1-yl)acetic acid:

To a solution of methyl 2-(2-(2-benzamidoethyl)-1H-benzo[d]imidazol-1-yl)acetate (1.4g, 4.15 mmol) in Tetrahydrofuran (THF) (30 mL) was added NaH (0.299 g, 12.4 mmol) followed by MeI (0.40 mL, 6.22 mmol) at 0ºC and was allowed to warm to rt and stirred for 21h. Water (20 mL) was added to the reaction mixture and was then extracted with ethyl acetate (50 mL). Then, the separated aqueous layer was acidified with 1N HCl to PH~3 and extracted with 10% MeOH in DCM (3 x 50 mL). The combined organics were dried over anhydrous Na_2_SO_4_, filtered and concentrated to obtain the crude product (800mg) which was carried over to the next step without any further purification. MS (ES^+^) m/z 338 (MH^+^), LCMS purity 54%.

Step-4: Preparation of N-methyl-N-(2-(1-(2-(naphthalen-2-ylamino)-2-oxoethyl)-1H-benzo[d]imidazol-2-yl)ethyl)benzamide Compound **32**:

To a solution of 2-(2-(2-(N-methylbenzamido)ethyl)-1H-benzo[d]imidazol-1-yl)acetic acid (200 mg, 0.593 mmol) in N,N-Dimethylformamide (DMF) (10 mL) was added EDC (170 mg, 0.889 mmol), HOBt (136 mg, 0.889 mmol) and TEA (0.124 mL, 0.889 mmol) at rt and was stirred for 30 min. Then, naphthalen-2-amine (102 mg, 0.711 mmol) was added and resulting mixture was stirred for 10h. The reaction was monitored by TLC(R_f_: 0.3, 5% MeOH in DCM), and upon completion of the reaction, water (50 mL) was added to the mixture. The resulting solution was extracted with EtOAc (2 x 50 mL) and the combined organics were dried over anhydrous Na_2_SO_4_, filtered and concentrated to obtain the crude material. Purification by silica gel (100-200 mesh) column chromatography using 2% of MeOH in DCM as an eluent afforded N-methyl-N-(2-(1-(2-(naphthalen-2-ylamino)-2-oxoethyl)-1H-benzo[d]imidazol-2-yl)ethyl)benzamide (48 mg, 0.10 mmol, 17% yield) as a brown solid. ^1^H NMR (DMSO-d_6_, 400 MHz): δ 2.93 (s, 3H), 3.03-3.23 (m, 2H), 3.90-3.94 (t, 2H), 5.03-5.26 (m, 2H), 7.18-7.26(m, 3H), 7.37-7.62 (m, 9H), 7.77-7.90 (m, 3H), 8.28 (m, 1H), 10.42-10.72 (m, 1H). MS (ES^+^) m/z 463 (MH^+^), LCMS purity 98%.

Preparation of N-(2-(1-(2-((3-isopropylphenyl)amino)-2-oxoethyl)-1H-benzo

[d]imidazol-2-yl)ethyl)-N-methylbenzamide Compound **33**:

Compound **33** was prepared according to the general procedure above to obtain N-(2-(1-(2-((3-isopropylphenyl)amino)-2-oxoethyl)-1H-benzo[d]imidazol-2-yl)ethyl)-N-methylbenzamide (50 mg, 0.105 mmol, 17% yield) as a pale yellow solid. ^1^H NMR (DMSO-d_6_, 400 MHz): δ 1.15-1.23 (d, 6H), 2.83-2.92 (m, 1H), 2.92 (s, 3H), 3.14-3.23 (m, 2H), 3.90-3.94 (t, 2H), 5.03-5.26 (m, 2H), 6.95-6.97(m, 1H), 7.17-7.41 (m, 9H), 7.47-7.62 (m, 2H), 10.42-10.47 (m, 1H). MS (ES^+^) m/z 455 (MH^+^), LCMS purity 95%.

Preparation of N-(2-(1-(2-(benzo[b]thiophen-5-ylamino)-2-oxoethyl)-1H-benzo[d] imidazol-2-yl)ethyl)-N-methylbenzamide Compound **34**:

Compound **34** was prepared according to the general procedure above to afford N-(2-(1-(2-(benzo[b]thiophen-5-ylamino)-2-oxoethyl)-1H-benzo[d]imidazol-2-yl)ethyl)-N-methylbenzamide (15 mg, 0.032 mmol, 5% yield) as an off white solid. ^1^H NMR (DMSO-d_6_, 400 MHz): δ 2.92 (S, 3H), 3.05-3.23 (m, 2H), 3.75-3.90 (m, 2H), 5.01-5.23 (m, 2H), 7.08-7.75 (m, 12H), 7.93-7.95 (m, 1H), 8.21-8.24 (m, 1H), 10.58-10.62 (m, 1H). MS (ES^+^) m/z 468 (MH^+^), LCMS purity 99%.

Preparation of N-(2-(1-(2-((3,4-dichlorophenyl)amino)-2-oxoethyl)-1H-benzo

[d]imidazol-2-yl)ethyl)-N-methylbenzamide Compound **35**:

Compound **35** was prepared according to the general procedure above to obtain the impure product. It was further purified by preparative HPLC to afford N-(2-(1-(2-((3,4-dichlorophenyl)amino)-2-oxoethyl)-1H-benzo[d]imidazol-2-yl)ethyl)-N-methylbenzamide (15 mg, 0.031 mmol, 5% yield) as a white solid. ^1^H NMR (DMSO-d_6_, 400 MHz): δ 2.93 (s, 3H), 3.03-3.23 (m, 2H), 3.74-3.94 (m, 2H), 4.97-5.21 (m, 2H), 7.18-7.61 (m, 11H), 7.95-7.97 (m, 1H), 10.77-10.81 (m, 1H). MS (ES^+^) m/z 480 (MH^+^), LCMS purity 98%.

Preparation of N-(2-(1-(2-(benzofuran-5-ylamino)-2-oxoethyl)-1H-benzo

[d]imidazol-2-yl)ethyl)-N-methylbenzamide Compound **36**:

Compound **36** was prepared according to the general procedure above to afford N-(2-(1-(2-(benzofuran-5-ylamino)-2-oxoethyl)-1H-benzo[d]imidazol-2-yl)ethyl)-N-methylbenzamide (32 mg, 0.070 mmol, 11% yield) as a pink solid. ^1^H NMR (DMSO-d_6_, 400 MHz): δ 2.93 (s, 3H), 3.03-3.23 (m, 2H), 3.74-3.94 (m, 2H), 4.97-5.21 (m, 2H), 6.92(d, 1H), 7.19-7.62 (m, 11H), 7.96-7.98 (m, 2H), 10.49-10.53 (m, 1H). MS (ES^+^) m/z 453 (MH^+^), LCMS purity 99%.

Preparation of N-(2-(1-(2-((3,4-dimethylphenyl)amino)-2-oxoethyl)-1H-benzo

[d]imidazol-2-yl)ethyl)-N-methylbenzamide Compound **37**:

Compound **37** was prepared according to the general procedure above to afford N-(2-(1-(2-((3,4-dimethylphenyl)amino)-2-oxoethyl)-1H-benzo[d]imidazol-2-yl)ethyl)-N-methylbenzamide (35 mg, 0.077 mmol, 13% yield) as an off white solid. ^1^H NMR (DMSO-d_6_, 400 MHz): δ 2.16 (s, 6H), 2.93 (s, 3H), 3.03-3.23 (m, 2H), 3.74-3.94 (m, 2H), 4.97-5.21 (m, 2H), 7.05-7.08 (d, 1H), 7.17-7.25 (m, 2H), 7.28-7.44 (m, 8H), 7.51-7.60 (m, 1H), 10.30-10.34 (m, 1H). MS (ES^+^) m/z 441 (MH^+^), HPLC purity 97%.
